# Improved salinity and dust stress tolerance in the desert halophyte *Haloxylon aphyllum* by halotolerant plant growth-promoting rhizobacteria

**DOI:** 10.3389/fpls.2022.948260

**Published:** 2022-08-03

**Authors:** Mahmood Najafi Zilaie, Asghar Mosleh Arani, Hassan Etesami, Mehri Dinarvand

**Affiliations:** ^1^Department of Environmental Sciences, Faculty of Natural Resources, Yazd University, Yazd, Iran; ^2^Department of Soil Science, University of Tehran, Karaj, Iran; ^3^Forests and Rangelands Research Department, Khuzestan Agricultural and Natural Resources Research and Education Center, Agricultural Research Education and Extension Organization (AREEO), Ahvaz, Iran

**Keywords:** *Bacillus pumilus*, *Halostachys belangeriana*, nutrients, PGPR, *Seidlitzia rosmarinus*, *Zhihengliuella halotolerans*

## Abstract

Because of global warming, desertification is increasing. One of the best strategies for combating desertification is reforestation of forests and biological operations of vegetation. However, events like soil salinity and dust storms, as the most important manifestations of desertification, prevent vegetation from settling in these areas. In this study, the effects of two halotolerant plant growth-promoting rhizobacterial strains, *Bacillus pumilus* HR and *Zhihengliuella halotolerans* SB, on physiological and nutritional status of the desert halophyte *Haloxylon aphyllum* under the stress of salinity (0, 300, and 600 mM NaCl) and dust (0 and 1.5 g m^−2^ month^−1^) were examined. Under dust application, the *Z. halotolerans* SB strain compared to the *B. pumilus* HR strain and the combination of these two bacterial strains improved the content of total chlorophyll (247 and 316%), carotenoid (94 and 107%), phosphorus (113 and 209%), magnesium (196 and 212%), and total dry biomass (13 and 28%) in *H. aphyllum* at salinity levels of 300 and 600 mM NaCl, respectively. Under conditions of combined application of dust and salinity, *B. pumilus* HR compared to *Z. halotolerans* SB and the combination of two strains at salinity levels of 300 and 600 mM NaCl, respectively, had better performance in increasing the content of iron (53 and 69%), calcium (38 and 161%), and seedling quality index (95 and 56%) in *H. aphyllum*. The results also showed that both bacterial strains and their combination were able to reduce the content of ascorbic acid, flavonoid, total phenol, proline, and malondialdehyde, and catalase activity, and ultimately improve the antioxidant capacity of *H. aphyllum*. This showed that the use of halotolerant rhizobacteria can stop the production of free radicals and thus prevent cell membrane damage and the formation of malondialdehyde under salinity and dust stress. The results of this study for the first time showed that halotolerant rhizobacteria can increase the seedling quality index of *H. aphyllum* under combined conditions of salinity and dust. The use of these bacteria can be useful in the optimal afforestation of *H. aphyllum* species in arid and semi-arid ecosystems.

## Introduction

Climate change is one of a number of variables that are considered to contribute to desertification. Desertification is land degradation in dry lands resulting from various factors, including both climatic variations and human activities (Safriel, 2009). Dust pollution, which is considered as one of the most important manifestations of desertification, is one of the main environmental challenges in most arid and semi-arid environments (Javanmard et al., 2019). Dust is defined as a collection of fine solid particles of natural or anthropogenic origin and is usually formed by disintegration processes (Arslan and Boybay, 1990). A set of atmospheric, geomorphic, and ecological processes and human activities are involved in the mechanism of production, transfer, and deposition of dust particles (Reheis and Urban, 2011). The major sources of soil dust include arid and semi-arid regions, particularly subtropical latitudes where the great deserts are located, including Africa, Middle East, Southwest Asia, central Australia, Mongolia, and parts of Europe and the Americas (Goudie, 2014).

It is known that tree plantation and forest belts can provide a cost-effective and eco-friendly solution to mitigate dust pollution (Javanmard et al., 2019; Naseri and Shakeri, 2019). However, dust particles directly affect plants by deposition on aerial parts or indirectly by altering the chemical characteristics of soil (Maletsika et al., 2015). Dust can cause serious damage to plant tissues by introduction of salt to land and soil salinization (Stefanski and Sivakumar, 2009). Dust deposition on plants usually affects the quantity and quality of light that reaches plant surfaces and elevates leaf temperature (Meravi et al., 2021). Furthermore, accumulation of dust particles on leaves may lead to stomata blocking, which interferes with gas exchange. All of the above changes can adversely affect leaf physiological and biochemical traits and negatively impact plant growth and productivity (Maletsika et al., 2015). The intensity of dust pollution on plants depends on its chemical and physical properties, duration and frequency of occurrence of dust events, leaf morphology, and species tolerance to such stress. Also, climatic characteristics such as temperature, relative humidity, wind speed, and precipitation events influence the intensity of dust stress on plants (Siqueira-Silva et al., 2016).

Approximately two-thirds of Iran's area is located in arid and semi-arid regions. Due to the lack of rainfall in arid and desert areas of Iran and consequent lack of biomass, increased vegetation productivity by cultivation of compatible plants can increase the ecosystem capacity and improve soil quality in these areas (Bazgeer et al., 2019). Studies show that rehabilitation of desert land in Iran by planting halophyte plants, especially *Haloxylon aphyllum*, has a high potential to increase carbon sequestration and control desertification (Loni et al., 2018; Taati et al., 2019).

It has also been reported that the morphological and physiological structures of halophytic plant species including *H. aphyllum*, as the dominant species in desert areas, have been negatively affected by salinity and dust stress (Mosleh-Arany et al., 2011; Heydarnezhad, 2014; Javanmard et al., 2019; Komaresofla et al., 2019). In a previous study, the application of aeolian dust on *H. aphyllum* caused a significant reduction in the content of chlorophyll a, chlorophyll b, carotenoid, total soluble sugars, and total nitrogen sugars of this plant (Heydarnezhad, 2014). Leaves constitute the most sensitive plant organ and are highly exposed to dust stress (Siqueira-Silva et al., 2016). When exposed to dust, leaves experience physiological changes (i.e., change in pH and relative water, total chlorophyll, and ascorbic acid content) before exhibiting visible damage symptoms (Hayat et al., 2012). Excess of dust may also lead to stomatal closure and thus can cause decreased photosynthesis (McDowell et al., 2008; Van Der Molen et al., 2011). Previous studies on dust-stressed plants have documented that dust deposition can influence plant vegetative growth, the ionic composition of tissues, and foliar temperature (Drack and Vázquez, 2018) because of change in leaf surface optical properties (Prajapati, 2012).

Salinity stress is one of the most common stresses in these areas that restrict the growth and production of plants (Etesami and Beattie, 2018; Kibria and Hoque, 2019) including halophytes (Komaresofla et al., 2019; Hidri et al., 2022). The direct effects of salt on plant growth involve nutrient imbalance caused by loss of control on nutrient uptake and/or transport to the shoot leading to ion deficiencies. The main reason for these nutrient deficiencies can be related to the abundant presence of ions, like Na^+^ and Cl^−^, in the soil solution. Abundance of these soluble ions can decrease the activity of other essential elements in the soil and can lead to reduction in accessibility and uptake of some elements by plants (Munns, 2002). Changes in morphological traits of salinity-stressed plants include decrease in germination, plant growth, and biomass (Kibria and Hoque, 2019; Azarmi-Atajan and Sayyari-Zohan, 2020) and changes in plant physiological and biochemical traits including decreased chlorophyll content, increased production of reactive oxygen species (ROS), disruption of the production of antioxidant enzymes and changes in their activity, degradation of cell wall proteins, and production of osmolytes (Bensidhoum et al., 2019). These negative effects are well recognizable in the form of changes in ionic content and plant growth-related properties and morphological traits (Kumar and Verma, 2018).

One of the biological solutions to deal with salinity stress is the use of halotolerant (high salinity resistant) plant growth-promoting bacteria (PGPB), which can improve the growth and yield of plants under stress conditions (Etesami and Maheshwari, 2018; Etesami and Glick, 2020; Amini Hajiabadi et al., 2021). The use of PGPB is also known as a possible strategy to increase rangeland forage production (Hungria et al., 2021; Zilaie et al., 2022). Halotolerant PGPB improve plant growth and yield by production of plant hormones, siderophore, and 1-aminocyclopropane-1-carboxylic acid (ACC) deaminase, nitrogen biological fixation, solubilization of insoluble phosphates and potassium-bearing minerals, and control of plant pathogens (Etesami and Alikhani, 2019; Etesami and Glick, 2020). It is well-known that halophytic plants or the rangeland plants that grow under harsh conditions harbor halotolerant PGPB with multiple plant growth-promoting (PGP) traits (Etesami and Beattie, 2018; Etesami and Glick, 2020). For example, in previous studies, halotolerant PGPB such as *Bacillus pumilus, Pseudomonas, Microbacterium, Zhihengliuella halotolerans*, and *Staphylococcus* with PGP traits from the halophytes *Suaeda* sp., *Salicornia* sp., *Halostachys belangeriana*, and *Seidlitzia rosmarinus* were isolated (Komaresofla et al., 2019; Alishahi et al., 2020; Amini Hajiabadi et al., 2021). In addition, these bacteria modulate and regulate the physiological and biochemical responses of plants and increase plant tolerance to salinity stress and, consequently, plant survival under stressful conditions (Del Carmen Orozco-Mosqueda et al., 2020; Amini Hajiabadi et al., 2021). The effective role of PGPB in increasing the growth and yield of plants, especially forage plants, has been shown by several researchers (Santos et al., 2019; Mamédio et al., 2020). For example, Mohmmad Abadi et al. (2021) used PGPB to improve the vegetative traits of *H. aphyllum* seeds. They showed that *Azotobacter* caused a significant increase in the root length, seedling growth, shoot fresh weight, and root fresh weight of *H. aphyllum* compared to the control. In another study, *Glutamicibacter* sp. and *Pseudomonas* sp. alleviated high salinity (600 mM NaCl) impact on the halophyte *Suaeda fruticosa* by modulating antioxidant defense (e.g., the activity of antioxidant enzymes of superoxide dismutase, catalase, ascorbate peroxidase, and glutathione reductase and decrease in malondialdehyde) and soil biological activity (Hidri et al., 2022). Some beneficial rhizobacteria that have attracted more attention because of their ability to communicate with plants in desert areas are the strains of *Bacillus pumilus* and *Zhihengliuela halotolerans*, whose positive effects on plant growth have also been proven (Jha et al., 2012; Ansari et al., 2019; Amini Hajiabadi et al., 2021).

Although the effect of various halotolerant PGPB on morpho-physiological, biochemical, and ionomic reactions of plants to salt stress has been reported, to date, there have been no reports of the impact of salt-tolerant PGPB on morpho-physiological, biochemical, and ionomic responses of halophytic plants under salinity and dust stress. It is still unclear how halotolerant PGPB interact with each other and affect halophytic plants under salinity and dust conditions. Thus, here, we report on the effects of two halotolerant rhizobacterial strains, *B. pumilus* HR, which has been previously isolated from halophytic plants (Amini Hajiabadi et al., 2021), as single and co-inoculation on morpho-physiological, biochemical, and ionomic properties of *H. aphyllum* under the stress of salinity and dust. In the study of Amini Hajiabadi et al. (2021), the two rhizobacterial strains could significantly improve the biochemical and morpho-physiological status of wheat plant and assist the plant to tolerate salt stress compared to un-inoculated wheat plants. Increased knowledge of halophytic plant–halotolerant PGPB interactions would enable for a better comprehension of their role during plant growth and development and translation into boosted biomass production and microbe-assisted phyto-technologies. The research questions of this study were (i) can these bacteria increase the growth of the halophyte *H. aphyllum* under saline and dust conditions and (ii) do the combined use of these two rhizobacterial strains have a greater effect on the growth of the halophyte *H. aphyllum* or their separate inoculation? Our hypothesis was that the effects of single and combined use of effective halotolerant rhizobacterial strains isolated from different halophytic plants on increased *H. aphyllum* plant growth under salinity and dust stress conditions would be different. According to our information, this is the first study to survey the effect of halotolerant rhizobacterial strains on the *H. aphyllum* plant, and it is also the first study to examine the impact of PGPB on plant dust stress tolerance. Such knowledge gaps highlight the need to investigate the potential of halotolerant PGPB in improving the tolerance of halophytic plants to dust stress in desert areas. The results of this study can be useful in optimal afforestation of *H. aphyllum* species and promotion of carbon dioxide sequestration in arid and semi-arid ecosystems under salt and dust stress.

## Materials and methods

### Halotolerant bacterial strains and inoculum preparation

In this study, two halotolerant rhizobacterial strains, *B. pumilus* HR (accession number: MW295357), previously isolated from the halophyte *Halostachys belangeriana*, and *Z. halotolerans* SB (accession number: MW295355), previously isolated from the halophyte *Seidlitzia rosmarinus* (Amini Hajiabadi et al., 2021), were used. How to isolate, identify, and determine the PGP traits and salinity resistance of these strains were reported in our previous study (Amini Hajiabadi et al., 2021). In a pretest, it was found that the two strains were able to colonize the roots of *H. aphyllum* in the presence and absence of salinity stress (data not shown). For preparing the bacterial inoculum, two bacterial strains were grown in a nutrient broth (NB) culture medium separately until they both reached the late exponential-phase (3 × 10^8^ cells ml^−1^) at 28 ± 2°C. Then, the bacterial cells were washed twice and re-suspended in sterile distilled water. Since the two bacterial strains were supposed to be inoculated into *H. aphyllum* together, we also showed in an *in vitro* evaluation, according to Etesami et al. (2014), that the two strains did not have any antagonistic effects on each other's growth.

### Experimental setup and treatments

To evaluate the effect of *B. pumilus* HR and *Z. halotolerans* SB on improved tolerance of the halophyte *H. aphyllum* to salinity and dust stress, a plant growth assay was carried out in a completely randomized design with factorial arrangement (4 × 3 × 2) with three replications in a research greenhouse located at Department of Environmental Engineering, Yazd-University, Yazd, Iran. The experimental treatments included: (a) halotolerant rhizobacterial strain factor at four levels: (i) seedlings non-inoculated with rhizobacterial strains (control), (ii) seedlings inoculated with *B. pumilus* HR, (iii) seedlings inoculated with *Z. halotolerans SB*, and (iv) seedlings co-inoculated with the HR and SB strains; (b) salinity stress factor at three levels: 0, 300, and 600 mM NaCl; and (c) dust factor at two levels: 0 and 1.5 g m^−2^ month^−1^.

Three-month-old seedlings of *H. aphyllum* were obtained from the Propagation Center of Rangeland Plants, Agricultural and Natural Resources-Research Center (ANRRC), Yazd, Iran. For bacterial inoculation, the roots of *H. aphyllum* seedlings were immersed in each bacterial suspension (3 × 10^8^ cells ml^−1^) for 2 h or in sterile water as un-inoculated control at room temperature. The seedlings that were to be co-inoculated with both strains of the bacteria were immersed in equal volumes of the suspension of the two bacterial strains. After inoculation, the inoculated or non-inoculated *H. aphyllum* seedlings with the same size and shape were transferred to plastic pots (40 × 25 cm) with drainage holes filled with 3 kg non–sterile dry soil (three units of soil without humus and one unit of blown sand) passed through a 4–mm sieve (one sapling per each pot). The soil used in this assay had 28.2% clay, 12% silt, and 59.2% sand with soil texture of sandy clay loam, pH 7.61, electrical conductivity (EC) 2 dS m^−1^, organic carbon 1.8 g kg^−1^, total N 0.2 g kg^−1^, NH_4_OAc–K 368 mg kg^−1^, Olsen-P 15 mg kg^−1^; Ca 1.8 meq kg^−1^, SO${}_{4}^{2-}$, 48 meq kg^−1^, Na 2.15 meq kg^−1^, and calcium carbonate (CaCO_3_) equivalent 32.5%. These properties were measured according to a previous method (Okalebo et al., 2002). Thirty days after planting the *H. aphyllum* seedlings, salinity and dust treatments were applied using irrigation water and a dust simulator (Dustin-mizer Model 1212 Includes Deflector, United States), respectively, according to the corresponding treatments. Salinity treatments were applied gradually to prevent shock to the *H. aphyllum* seedlings. In addition, the EC of the springlet of pots was measured once a week to ensure the desired salinity level, and in case of excessive increment in the desired salinity level, leaching with water was carried out. Dust was applied at 1.5 g m^−2^ month^−1^ once a week for 5 months based on the results of Ahmadi Foroushani et al. (2021). After pouring the dust into the valve of the dust simulator, the amount of dust (g m^−2^) was controlled using a trap with dimensions of 1.6 × 2.3 m^2^. When applying dust, all the control plants were moved outside the greenhouse so that dust does not settle on them. The dust used had 2.01% gravel, 22.24% sand, 43.74% silt, 21.83% clay; Ca, 22.70%; Mg, 2.42%; Fe, 4.71%; Na, 2.37%; Al, 4.08%; K, 0.79; Zn, 52.90 mg kg^−1^; Cu, 15.80 mg kg^−1^; Co, 12.70 mg kg^−1^; U, 1.96 mg kg^−1^; Cd, 0.60 mg kg^−1^; Ni, 94.00 mg kg^−1^; V, 73.40 mg kg^−1^; Ba, 231.00 mg kg^−1^; Cr, 116 mg kg^−1^; Pb, 20.23 mg kg^−1^; and pH, 7.6. The plants were grown in a glasshouse at 25 ± 2°C with a photoperiod of 16-h light and 8-h darkness and 60% relative humidity. At the end of the experiment period (150 days after planting in pots), some morpho-physiological, biochemical, and ionomic traits of *H. aphyllum* leaf were measured.

### Measurements

#### Determination of total chlorophyll

To extract chlorophyll a, chlorophyll b, and total chlorophyll, 0.5 g fresh leaf samples were taken and homogenized with 10 ml of 80% acetone. The homogenized samples were centrifuged at 4,000 rpm for 10 min at 4°C. The supernatants were separated from the mixture and collected in a cuvette for further use. To measure chlorophyll a, chlorophyll b, and total chlorophyll, the spectrophotometric method (to measuring absorbance of the extracts at 663.2 and 646.8 nm) proposed by Lichtenthaler (1987) was employed using a UV-VIS spectrophotometer (Varian Cary 50; Varian GmbH, Darmstadt, Germany). The concentrations of photosynthetic pigments present in the extracts were estimated using the following equations:


$$\begin{array}{l} Total\;chlorophyll\text{ = [12}{\text{.5 (A}}_{\text{663.2}}\text{) }-\text{2}{\text{.79 (A}}_{\text{646.8}}\text{)] }\\ \text{ +[21}{\text{.5A(}}_{\text{646.8}}\text{) }-\text{5}{\text{.1A (}}_{\text{663.2}}\text{)].} \end{array}$$

#### Determination of carotenoid content

A carotenoid analysis was performed as described by Lichtenthaler (1987). About 25 mg of plant material was mixed with 5 ml of 80% (*v*/*v*) acetone solution, and the mixture was vortexed followed by sonication for 30 min. After centrifugation for 10 min at 6,000 rpm, the absorbance of the supernatant was measured at 450, 646, and 663 nm to determine the total carotenoids, and the results were expressed in mg g^−1^ FW.

#### Determination of anthocyanin content

For determination of anthocyanin content, frozen tissue samples (100 mg) were soaked immediately in 10 ml of acidified methanol [methanol: HCl 99:1 (*v*/*v*)]. Tissues were crushed using a glass pestle and kept at 25°C for 24 h in the dark. The extract was then centrifuged at 4,000 *g* for 5 min at room temperature, and the absorption at 550 nm of the supernatant was read with a UV-VIS spectrophotometer (Varian Cary 50; Varian GmbH, Darmstadt, Germany). For calculation of the amount of anthocyanins, the extinction coefficient of 33,000 M^−1^ Cm^−1^ was used (Wagner, 1979). Anthocyanin concentration was expressed in μmol g^−1^ FW of tissue.

#### Determination of ascorbic acid content

Ascorbic acid content was determined based on the spectrophotometric method according to a previous study (Mukherjee and Choudhuri, 1983). Briefly, the sample (100 mg) was extracted with 10 ml of 1% metaphosphoric acid for 45 min at room temperature and filtered through Whatman No. 4 filter paper. The filtrate (1 ml) was then mixed with 9 ml of 2,6-dichlorophenolindophenol, and the absorbance was measured within 30 min at 520 nm against a blank. The content of ascorbic acid was calculated on the basis of the calibration curve of authentic L-ascorbic acid. The content of ascorbic acid was expressed in mg g^−1^ FW of tissue.

#### Determination of flavonoid content

To determine the content of flavonoid, 0.1 g of leaf tissue was extracted in a 15-ml glass centrifuge tube containing 10 ml of acidified ethanol [ethanol: acetic acid, 99:1 (*v/v*)]. The samples were gently boiled in a water bath at 80°C for 10 min and brought up to volume. Absorbance was measured at a wavelength of 315 nm with a UV-VIS spectrophotometer (Varian Cary 50; Varian GmbH, Darmstadt, Germany) (Krizek et al., 1998). The content of flavonoid acid was expressed in μmol g^−1^ FW of tissue.

#### Determination of total phenol

The content of total phenols was determined using the Folin-Ciocalteu reagent method (Hayouni et al., 2007). According to this method, 100 μl of the extract with a concentration of 1 mg ml^−1^ was added to 500 μl of the Folin-Ciocalteu reagent and 1.5 ml of 20% sodium bicarbonate solution, and incubated for 120 min at room temperature. The sample absorbance at 760 nm was read with a UV-VIS spectrophotometer (Varian Cary 50; Varian GmbH, Darmstadt, Germany). The standard curve was prepared with 50–500 mg L^−1^ gallic acid solution in methanol. The content of total phenols was expressed as percentage.

#### Determination of proline content

The proline content of plant leaves was determined according to a previous method (Bates et al., 1973). From each plant, about 100 mg of leaves were homogenized in 1.5 ml of 3% sulfosalicylic acid. After centrifugation of the extracts at 4,000 rpm, 100 μl of the supernatant was transferred to new tubes and mixed with 2 ml of glacial acetic acid and 2 ml of ninhydrin. The mixture was incubated in a water bath at 100°C for 1 h. After this period, the tubes were placed in ice, and 1 ml of toluene was added to the cooled tubes. The absorbance of the chromophore solution was measured at 520 nm with a UV-VIS spectrophotometer (Varian Cary 50; Varian GmbH, Darmstadt, Germany). The content of proline in the samples was then extrapolated from a calibration line obtained measuring the absorbances of proline solutions of known concentration. The content of proline was expressed in μg g^−1^ FW of tissue.

#### Water soluble sugar content

The method described by Irigoyen et al. (1992) was used to measure soluble sugar content. For this purpose, 10 ml of 70% ethanol was added to 0.1 g of plant dry sample and stored in a refrigerator for 1 week. After this period, 0.5 ml of the supernatant was taken, and its volume was brought to 2 ml with distilled water. Then, 1 ml of 5% phenol was added to it and mixed well, and then 5 ml of concentrated sulfuric acid was added to it. A yellow solution was obtained and changed color over time and tended to become light brown. After 30 min, the absorbance of the samples was read using a spectrophotometer (Analytik Jena 210, Germany) at 485 nm and room temperature. Using the standard glucose curve, soluble sugar content in mg g^−1^ fresh weight was assessed.

#### Determination of leaf extract pH

Leaf extract pH was determined by the protocol of Tak and Kakde (2017). For each treatment, four samples of 0.5 g of fresh leaves were crushed and homogenized in 50 ml deionized water, after which the mixture was centrifuged at 7,000×g for 10 min. The pH of the supernatant was measured using a digital pH meter (EYELA, Japan).

#### Determination of catalase activity

Extraction of fresh leaves (0.5 g) was performed according to Mukherjee and Choudhuri (1983). The extract was frozen in liquid nitrogen and then ground in a phosphate buffer (100 mM, pH 7). Homogenates were centrifuged at 4°C for 10 min at 15,000 g. The supernatant was kept at 4°C until used to measure the activity of peroxidase and catalase. An Aebi (1984) test was conducted to evaluate catalase (EC1.11.1.6) activity. The decline in absorbance recorded at 240 nm as an outcome of H_2_O_2_ consumption reveals enzyme activity. The activity of the enzymes was expressed in U mg^−1^ protein.

#### Determination of relative water content

For determination of relative water content (RWC), each leaf was weighed on a digital scale (GF-300; A&D Company, Tokyo, Japan) with an accuracy of 0.001 g (F_W_) and then placed in Falcon tubes completely filled with distilled water. The tubes were left in the dark at 4°C for 24 h. After this period, the leaves were removed from water and placed on absorbent paper to remove excess water, and turgid weight (T_w_) was determined. Afterward, the leaves were dried at 70°C until stabilization, and dry weight was determined (D_w_). Leaf RWC was calculated using the following equation and expressed as a percentage (Ritchie et al., 1990):


$$\begin{array}{l} \text{RWC }\left(\text{\%}\right)=\left[\left(\text{Fw}-\text{Dw}\right)/\left(\text{Tw}-\text{Fw}\right)\right]\times \;100. \end{array}$$

#### Determination of DPPH radical scavenging capacity

The 2,2-diphenyl-1-picrylhydrazyl (DPPH) method was used to determine the free radical scavenging activity of each sample (Brand-Williams et al., 1995). About 1.4 ml DPPH solution (0.0062 g 100 ml^−1^ MeOH) was mixed with 0.2 ml of the tested samples dissolved in water at different concentrations (sample concentrations corresponding to 0.5–50 mg of plant material per 1 ml final solution, prepared by diluting the original plant extracts with the extraction solvent). The reaction mixture was shaken and incubated in the dark at room temperature for 30 min. Absorbance (A) was measured at 536 nm against the blank (UV-VIS spectrophotometer). Controls were prepared as for the test group except that the antioxidant solution was replaced with the corresponding extraction solvent. Inhibition of the DPPH radical by the sample was calculated according to the following formula:


$$\begin{array}{l} \text{DPPH scavenging activity }\left(\text{\%}\right)=\left[\text{A}0-\text{A}1/\text{A}1\right]\times \;100, \end{array}$$

where A0 is the absorbance of the control and A1 is the absorbance of the sample. Free radical scavenging activity was expressed as the percentage of DPPH decrease.

#### Determination of malondialdehyde content

The content of malondialdehyde in samples of plant tissue was determined with the thiobarbituric acid method according to a previous study (Stewart and Bewley, 1980). Briefly, 0.5 g of fresh leaf sample was homogenized with 5 ml of 0.1% trichloroacetic acid. The homogenates were centrifuged for 5 min at 4,000 rpm and 4°C. Then, aliquots of 1 ml of the supernatant were transferred to falcon tubes, and 4 ml of 20% trichloroacetic acid solution containing 0.5% thiobarbituric acid was added to the tubes. The tubes were placed in a water bath, at 95°C for 30 min. After cooling in ice, the tubes were centrifuged for 10 min at 4,000 rpm and 4°C. The specific and non-specific absorbance of the supernatant was measured at 532 and 600 nm, respectively. Distilled water was used as blank for zeroing the absorbance of the UV-VIS spectrophotometer (Varian Cary 50; Varian GmbH, Darmstadt, Germany). Malondialdehyde content was calculated subtracting the nonspecific absorbance at 600 nm and using the molar extinction coefficient ε = 155 mM^−1^ cm^−1^. The content of malondialdehyde was expressed in nmol g^−1^ FW of tissue.

#### Determination of nutrient content

Dried powdered leaves were digested in a diacid mixture of HNO_3_:HClO_4_ (4:1) for P, K, and Na analyses (Waling et al., 1989). Phosphorus concentration was tested in the digested sample. Sodium and K concentrations in the digest were analyzed using a flame photometer (Jenway, England). The concentration of Mg, Ca, and Fe was also determined by atomic absorption spectroscopy (nov AA 300) (Waling et al., 1989).

#### Determination of total dry biomass and seedling quality index

Shoots (stem and leaves) and roots were washed with distilled water; thereafter, they were blotted dry gently on a paper towel and dried for 48 h at 70°C for determination of total dry biomass. The measurements were conducted on a digital scale (GF-300; A&D Company, Tokyo, Japan). Plant height was measured using a ruler, and stem base diameter was also measured using a digital caliper. Finally, seedling quality index was calculated with the following equation (Dickson et al., 1960):


$$\begin{array}{l} \text{Seedling quality index = Total dry biomass in g/}[(\text{Plant}\\ \text{ height incm/Stem base diameter in mm})\text{+}(\text{Shoot dry}\\ \text{ weight in g/Root dry weight in}\;\text{g})]. \end{array}$$

### Statistical analysis

After confirming the normality of the data by Kolmogorov–Smirnov test, experimental data were analyzed by three-way analysis of variance (ANOVA) using the SAS v.9.1 (SAS Institute Inc., Cary, NC) statistical analysis software. The presented data were means of three replicates per treatment ± standard error (SE). A comparison of means for the individual treatments was made at the 5% probability level by Duncan's new multiple range test. A principal component analysis (PCA) based on the Pearson method was conducted using the PAST 3.0 software to determine the relationship between the measured plant parameters with bacterial strains, salinity stress, and dust stress.

## Results

### Effect of bacterial strains on leaf total chlorophyll content

**Figure 1:**
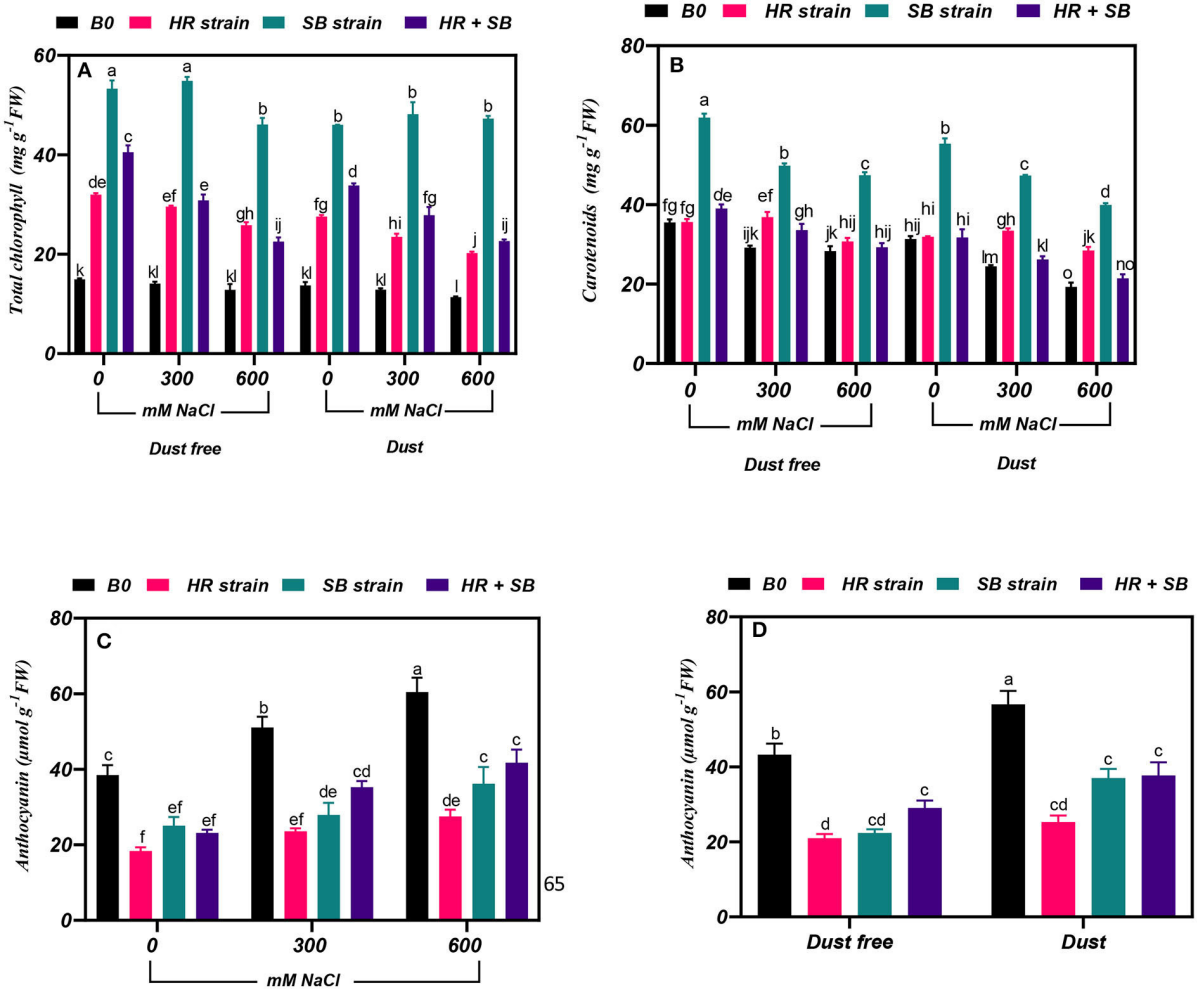
Triple/dual interactions of treatments of halotolerant bacterial strains (*Bacillus pumilus* HR and *Zhihengliuella halotolerans* SB), salinity levels (irrigation with saline water), and dust on the content of **(A)** total chlorophyll, **(B)** carotenoids, and **(C,D)** anthocyanin of *Haloxylon aphyllum* seedlings grown under greenhouse conditions for 150 days. Means ± SE (*n* = 3) followed by the same letters are not significantly different according to Duncan's new multiple range test at *P* < 0.05.

The results showed that under non-saline conditions, the inoculation of *H. aphyllum* seedlings with HR strain, SB strain, and the combination of the two strains caused a significant increase in total chlorophyll by 114, 257, and 171%, respectively, under dust-free conditions and by 101, 236, and 142%, respectively, under dust application conditions. In addition, the HR and SB strains, and their combination increased the content of total chlorophyll in the plant by 110, 289, and 118% at the 300-mM NaCl level and by 101, 259, and 76% at the 600-mM NaCl level, respectively, under dust-free conditions. Under dust application conditions, the HR and SB strains, and their combination also increased the content of total chlorophyll in the plant by 82, 274, and 116% at the 300-mM NaCl level and by 78, 316, and 99% at the 600-mM NaCl level, respectively (Figure 1A).

### Effect of bacterial strains on carotenoid content

The results showed that under the bacteria-free and dust-free conditions, the content of carotenoids at 300 and 600 mM NaCl levels was decreased by 18 and 20%, respectively. At the 300 and 600 mM NaCl levels, the content of carotenoids was decreased by 22 and 38%, respectively, compared to the control by applying the dust treatment (Figure 1B). The dust treatment alone (without bacteria and salinity stress) increased the carotenoid content by 12%. Inoculation with the SB strain and the combination of the two strains caused a significant increase in carotenoid content in the plant by 74 and 10% under the non-saline and dust-free conditions, respectively, and under non-saline conditions this bacterial strain caused a 76% increase in carotenoid in plants treated with dust. The HR and SB strains, and their combination increased the content of carotenoid by 26, 71, and 15%, respectively. At the 600-mM NaCl level, the SB strain also increased the content of carotenoids by 76% under dust-free conditions, respectively. The HR and SB strains increased the content of carotenoids by 37 and 94% at the 300-mM NaCl level and by 47 and 107% at the 600-mM NaCl level, respectively, under dust application conditions (Figure 1B).

### Effect of bacterial strains on anthocyanin content

Under bacterial strain free and dust-free conditions, the content of anthocyanin was increased by 33 and 57% at the 300- and 600-mM NaCl levels, respectively, compared to the control (Figure 1C). The dust treatment alone (without bacteria and salinity) increased the content of anthocyanin by 31% (Figure 1D). By increasing salinity concentration to 600 mM NaCl, the content of anthocyanin was increased by 65% compared to the control. Under non-saline conditions, inoculation with the HR and SB strains, and their combination significantly reduced the content of anthocyanins by 52, 48, and 33% under dust-free conditions and by 55, 35, and 33% under dust application conditions, respectively. In addition, under dust-free conditions, the HR and SB strains, and their combination decreased the content of anthocyanin by 54, 45, and 31% at the 300-mM NaCl level and by 53, 40, and 31% at the 600-mM NaCl level, respectively (Figure 1D).

### Effect of bacterial strains on ascorbic acid content

**Figure 2:**
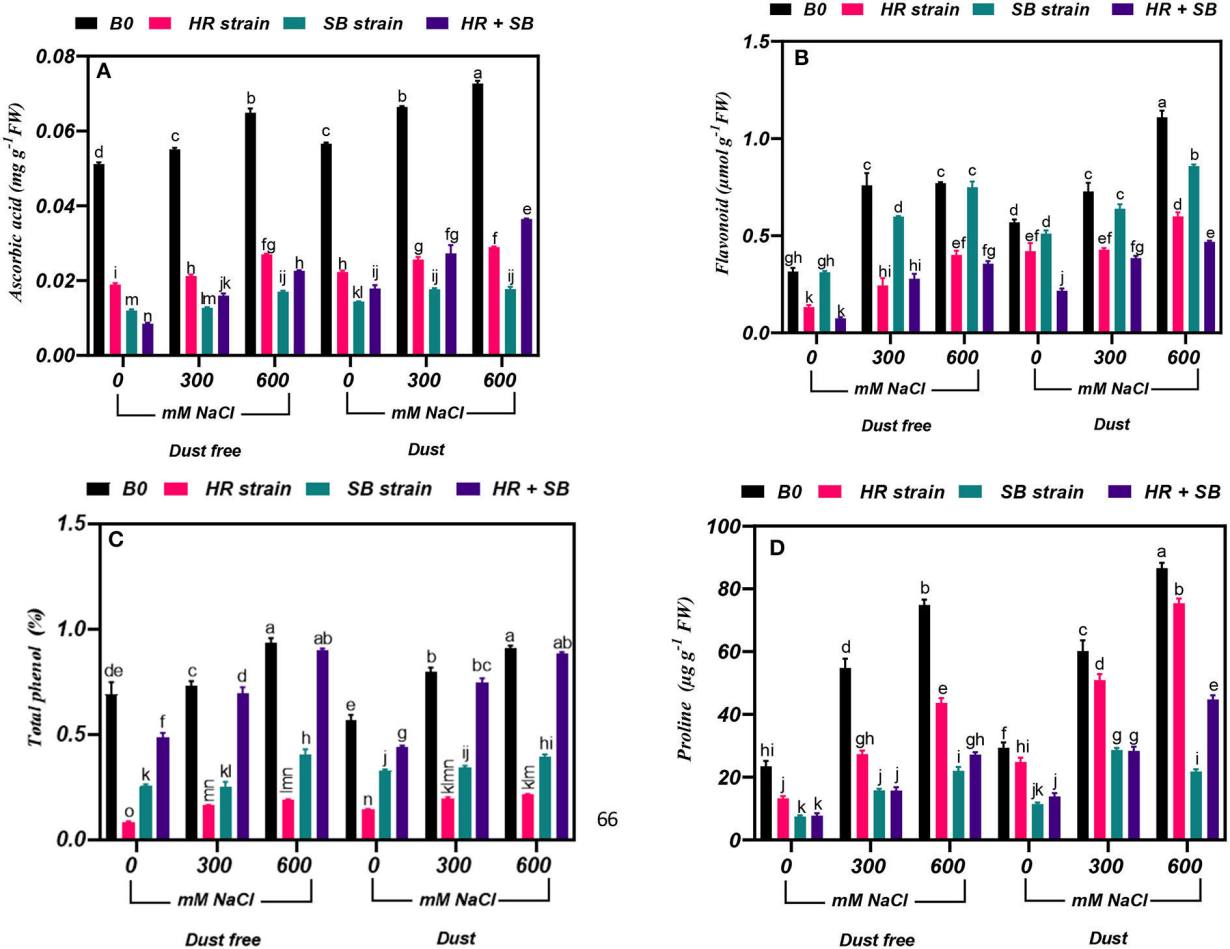
Triple/dual interactions of treatments of halotolerant bacterial strains (*B. pumilus* HR and *Z. halotolerans* SB), salinity levels (irrigation with saline water), and dust on the content of **(A)** ascorbic acid, **(B)** flavonoid, **(C)** total phenol, and **(D)** proline of *H. aphyllum* seedlings grown under greenhouse conditions for 150 days. Means ± SE (*n* = 3) followed by the same letters are not significantly different according to Duncan's new multiple range test at *P* < 0.05.

The results showed that under the bacteria-free and dust-free conditions, the content of ascorbic acid was increased by 8 and 30% at the 300- and 600-mM NaCl levels, respectively, and by 17 and 28% under dust application conditions, respectively. The dust treatment alone (without bacteria and salinity) increased the content of ascorbic acid by 11%. The HR and SB strains, and their combination significantly reduced the ascorbic acid content in salt non-stressed plants by 63, 77, and 83% under dust-free conditions and by 60, 74, and 68% under dust conditions, respectively. Also, under dust-free conditions, the HR and SB strains, and their combination decreased the content of ascorbic acid by 62, 77, and 71% at the 300-mM NaCl level and by 58, 74, and 65% at the 600-mM NaCl level, respectively. In addition, under dust application conditions, the HR and SB strains, and their combination decreased the content of ascorbic acid by 61, 74, and 59% at the 300-mM NaCl level and by 60, 76, and 50% at the 600-mM NaCl level, respectively (Figure 2A).

### Effect of bacterial strains on flavonoid content

The results showed that under the bacteria-free and dust-free conditions, the flavonoid content was increased by 140 and 144% at the 300- and 600-mM NaCl levels, respectively, and by 28 and 95% under dust application conditions, respectively. The dust treatment alone (without bacteria and salinity) increased the flavonoid content by 80%. Under non-saline conditions, the inoculation with the HR strain and the combination of the two strains significantly reduced the flavonoid content by 58 and 76% under dust conditions and by 26 and 62% under dust conditions, respectively. Also, the HR and SB strains, and their combination reduced the content of flavonoids by 68, 21, and 63% at the 300-mM NaCl level, respectively. At the 600-mM NaCl level, the HR strain and the combination of the two strains also reduced the content of flavonoid by 48 and 54%, respectively, under dust-free conditions. Also, the HR strain and the combination of the two strains reduced the content of flavonoid by 41 and 47% at the 300-mM NaCl level, respectively, and at the 600-mM NaCl level, the HR and SB strains, and their combination decreased flavonoid content by 46, 23, and 58% compared to the control, respectively, under dust conditions (Figure 2B).

### Effect of bacterial strains on total phenol content

Under the bacteria-free and dust-free conditions, total phenol content was increased by 6 and 35% at the 300- and 600-mM NaCl levels, respectively, and by 41 and 61% under dust application conditions, respectively. Under non-saline conditions, the inoculation with the HR strain, SB strain, and the combination of the two strains significantly reduced total phenol content by 88, 63, and 30% under dust conditions and by 75, 42, and 22% under dust conditions, respectively. Also, HR strain, SB strain, and the combination of the two strains reduced total phenol content by 78, 66, and 5% at the 300-mM NaCl level, respectively. At the 600-mM NaCl level, the HR and SB strains also reduced total phenol content by 80 and 57%, respectively, under dust-free conditions. In addition, under dust application conditions, the HR and SB strains decreased total phenol content by 75 and 57% at the 300-mM NaCl level, respectively. At the 600-mM NaCl level, the HR and SB strain decreased total phenol content by 77 and 56% compared to the control under dust applications, respectively (Figure 2C).

### Effect of bacterial strains on proline content

Under the bacteria-free and dust-free conditions, proline content was increased by 133 and 219% at the 300- and 600-mM NaCl levels, respectively, and by 105 and 195% under dust application conditions, respectively. The dust treatment alone (without bacteria and salinity) increased proline content by 25%. Under non-saline conditions, the inoculation with the HR and SB strains, and the combination of the two strains significantly reduced proline content by 43, 68, and 67% under dust conditions and by 15, 61, and 53% under dust conditions, respectively. Also, under dust-free conditions the HR strain, SB strain, and the combination of the two strains decreased proline content by 50, 71, and 71% at the 300-mM NaCl level and by 42, 70, and 64% at the 600-mM NaCl level, respectively. In addition, under dust application conditions, the HR strain, SB strain, and the combination of the two strains decreased the content of proline by 15, 52, and 53% at the 300-mM NaCl level and by 13, 75, and 48% at the 600-mM NaCl level, respectively (Figure 2D).

### Effect of bacterial strains on soluble sugar content

**Figure 3:**
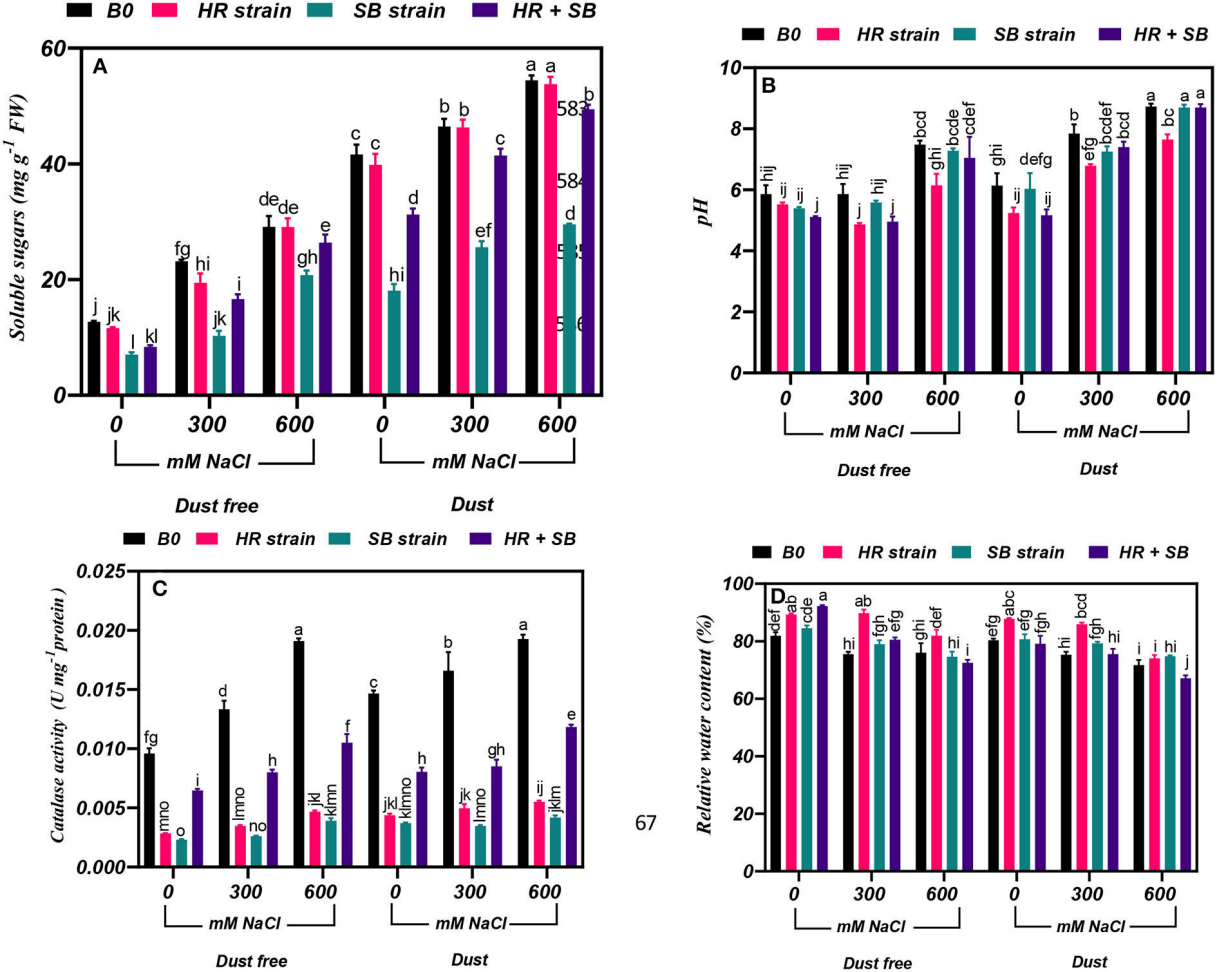
Triple/dual interactions of treatments of halotolerant bacterial strains (*B. pumilus* HR and *Z. halotolerans* SB), salinity levels (irrigation with saline water), and dust on the content of **(A)** soluble sugars, **(B)** pH, **(C)** catalase, and **(D)** relative water of *H. aphyllum* seedlings grown under greenhouse conditions for 150 days. Means ± SE (*n* = 3) followed by the same letters are not significantly different according to Duncan's new multiple range test at *P* < 0.05.

Under the bacteria-free and dust-free conditions, soluble sugar content was increased by 83 and 130% at the 300- and 600-mM NaCl levels, respectively, and by 12 and 31% under dust application conditions, respectively. The dust treatment alone (without bacteria and salinity) increased soluble sugar content by 228%. Under non-saline conditions, the inoculation with the SB strain and the combination of the two strains significantly reduced soluble sugar content by 45 and 34% under dust conditions and by 57 and 25% under dust conditions, respectively. Also, under dust-free conditions, the HR strain, SB strain, and the combination of the two strains decreased soluble sugar content by 16, 56, and 28% at the level of 300 mM NaCl, respectively. At the 600-mM NaCl level, the SB strain and the combination of these strains decreased soluble sugar content by 30 and 9% compared to the control under dust free applications, respectively. In addition, under dust application conditions, the SB strain and the combination of the two strains decreased soluble sugar content by 45 and 11% at the 300-mM NaCl level and by 45 and 9% at the 600-mM NaCl level, respectively (Figure 3A).

### Effect of bacterial strains on pH content

Under the bacteria-free and dust-free conditions, pH was increased by 28% at the 600-mM NaCl level, and it was also increased by 28 and 42% at the 300- and 600-mM NaCl levels, respectively, under dust application conditions. Under non-saline conditions, the inoculation with the HR strain and the combination of the two strains significantly reduced pH content by 15 and 16% under dust conditions. The combination of the two strains also reduced pH by 15% at the 300-mM NaCl level and by 18% at the 600-mM NaCl level under dust-free conditions. Also, at the 300- and 600-mM NaCl levels, the HR strain reduced pH by 13 and 12%, respectively, under dust application conditions (Figure 3B).

### Effect of bacterial strains on catalase activity

Under the bacteria-free and dust-free conditions, catalase activity was increased by 39 and 99% at the 300- and 600-mM NaCl levels, respectively, and by 13 and 31% under dust application conditions, respectively. The dust treatment alone (without bacteria and salinity) increased catalase activity by 53%. Under non-saline conditions, the inoculation with the HR strain, SB strain, and the combination of the two strains significantly reduced catalase activity by 70, 76, and 33% under dust-free conditions and by 70, 74, and 57% under dust conditions, respectively. Also, under dust-free conditions, the HR strain, SB strain, and the combination of the two strains decreased catalase activity by 74, 80, and 40% at the 300-mM NaCl level, and by 76, 79, and 45% at the 600-mM NaCl level, respectively. Under dust application conditions, the HR strain, SB strain, and the combination of the two strains decreased catalase activity by 70, 79, and 48% at the 300-mM NaCl level, and by 71, 78, and 39% at the 600-mM NaCl level, respectively (Figure 3C).

### Effect of bacterial strains on relative water content

Under the bacteria-free and dust-free conditions, relative water content was decreased by 8 and 7% at the 300- and 600-mM NaCl levels, respectively, and by 6 and 11% under dust application conditions, respectively. Under non-saline conditions, the inoculation with the HR strain and the combination of the two strains significantly increased relative water content by 9 and 13% under dust-free conditions. Under non-saline conditions, the inoculation with the HR strain increased relative water content by 9% under dust application conditions. Also, under dust-free conditions, the HR strain and the combination of the two strains increased relative water content by 19 and 7% at the 300-mM NaCl level, respectively. At the 600-mM NaCl level, the HR strain increased relative water content by 8% under dust-free conditions. Also, the HR strain at the 300-mM NaCl level increased relative water content by 14% compared to the control, but at the 600-mM NaCl level, the combination of the two strains reduced relative water content by 6% compared to the control (Figure 3D).

### Effect of bacterial strains on DPPH radical scavenging capacity

**Figure 4:**
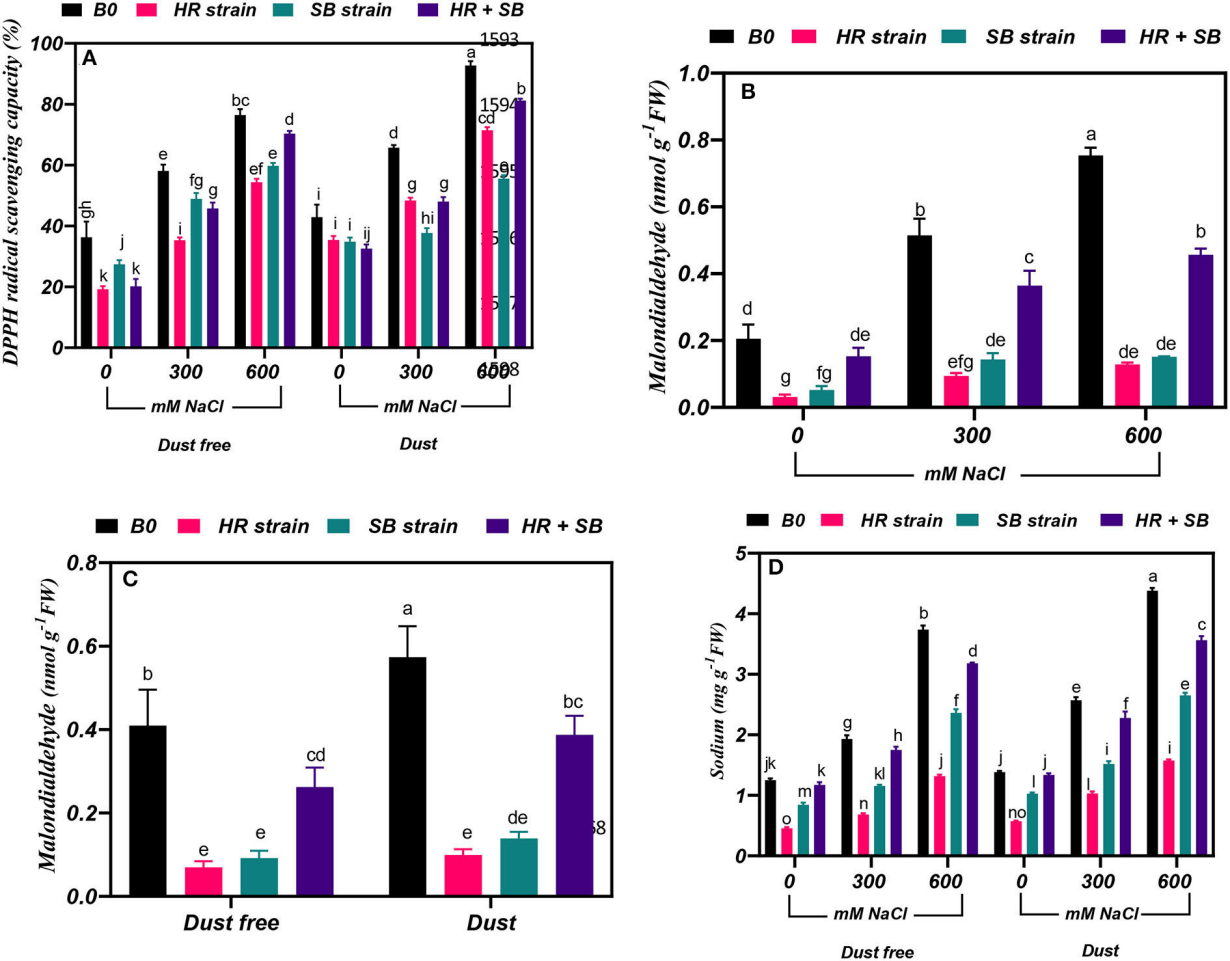
Triple/dual interactions of treatments of halotolerant bacterial strains (*B. pumilus* HR and *Z. halotolerans* SB), salinity levels (irrigation with saline water), and dust on **(A)** 2,2-Diphenyl-1-picrylhydrazyl (DPPH) radical scavenging capacity and the content of **(B,C)** malondialdehyde, and **(D)** sodium of *Haloxylon aphyllum* seedlings grown under greenhouse conditions for 150 days. Means ± SE (*n* = 3) followed by the same letters are not significantly different according to Duncan's new multiple range test at *P* < 0.05.

Under the bacteria-free and dust-free conditions, DPPH radical scavenging capacity was increased by 60 and 110% at the 300- and 600-mM NaCl levels, respectively, and by 53 and 116% under dust application conditions, respectively. The dust treatment alone (without bacteria and salinity) increased DPPH radical scavenging capacity by 18%. Under non-saline conditions, the inoculation with the HR strain, SB strain, and the combination of the two strains significantly reduced DPPH radical scavenging capacity by 47, 25, and 44%, respectively, under dust-free conditions. The HR strain, SB strain, and the combination of the two strains increased DPPH radical scavenging capacity by 39, 16, and 21% at the 300-mM NaCl level and by 28, 22, and 8% at the 600-mM NaCl level, respectively, under dust-free conditions. Under dust application conditions, the HR strain, SB strain, and the combination of the two strains increased DPPH radical scavenging capacity by 26, 43, and 27% at the 300-mM NaCl level and by 24, 40, and 12% at the 600-mM NaCl level, respectively (Figure 4A).

### Effect of bacterial strains on malondialdehyde content

Under the bacteria-free and dust-free conditions, malondialdehyde content was increased by 150 and 267% at the 300- and 600-mM NaCl levels, respectively (Figure 4B). The dust treatment alone (without bacteria and salinity) increased malondialdehyde content by 40% (Figure 4C). By increasing the salinity concentration to the 600-mM NaCl level, the amount of malondialdehyde was increased by 150% compared to the control. Under non-saline conditions, the inoculation with the HR strain, SB strain, and the combination of the two strains caused a significant decrease in malondialdehyde content by 83, 78, and 36%, respectively, under dust-free conditions. Also, the HR and SB strains significantly reduced malondialdehyde content by 82 and 75% under non-saline conditions and dust treatment application. Under dust-free conditions, the HR strain, SB strain, and the combination of the two strains decreased the content of malondialdehyde by 81, 72, and 30% at the 300-mM NaCl level and by 83, 80, and 39% at the 600-mM NaCl level compared to the control (Figure 4B).

### Effect of bacterial strains on sodium content

Under the bacteria-free and dust-free conditions, sodium content was increased by 54 and 198% at the 300- and 600-mM NaCl levels, respectively, and by 86 and 217% under dust application conditions, respectively. Under non-saline conditions, the inoculation with the HR and SB strains significantly reduced sodium content by 64 and 33%, respectively, under dust-free conditions and by 59 and 26%, respectively, under dust application conditions. Under dust-free conditions, the HR strain, SB strain, and the combination of the two strains decreased sodium content by 65, 40, and 9% at the 300-mM NaCl level and by 64, 37, and 15% at the 600-mM NaCl level compared to the control. Under dust application conditions, the HR strain, SB strain, and the combination of the two strains decreased sodium content by 60, 41, and 28% at the 300-mM NaCl level and by 64, 42, and 19% at the 600-mM NaCl level compared to the control (Figure 4D).

### Effect of bacterial strains on potassium content

**Figure 5:**
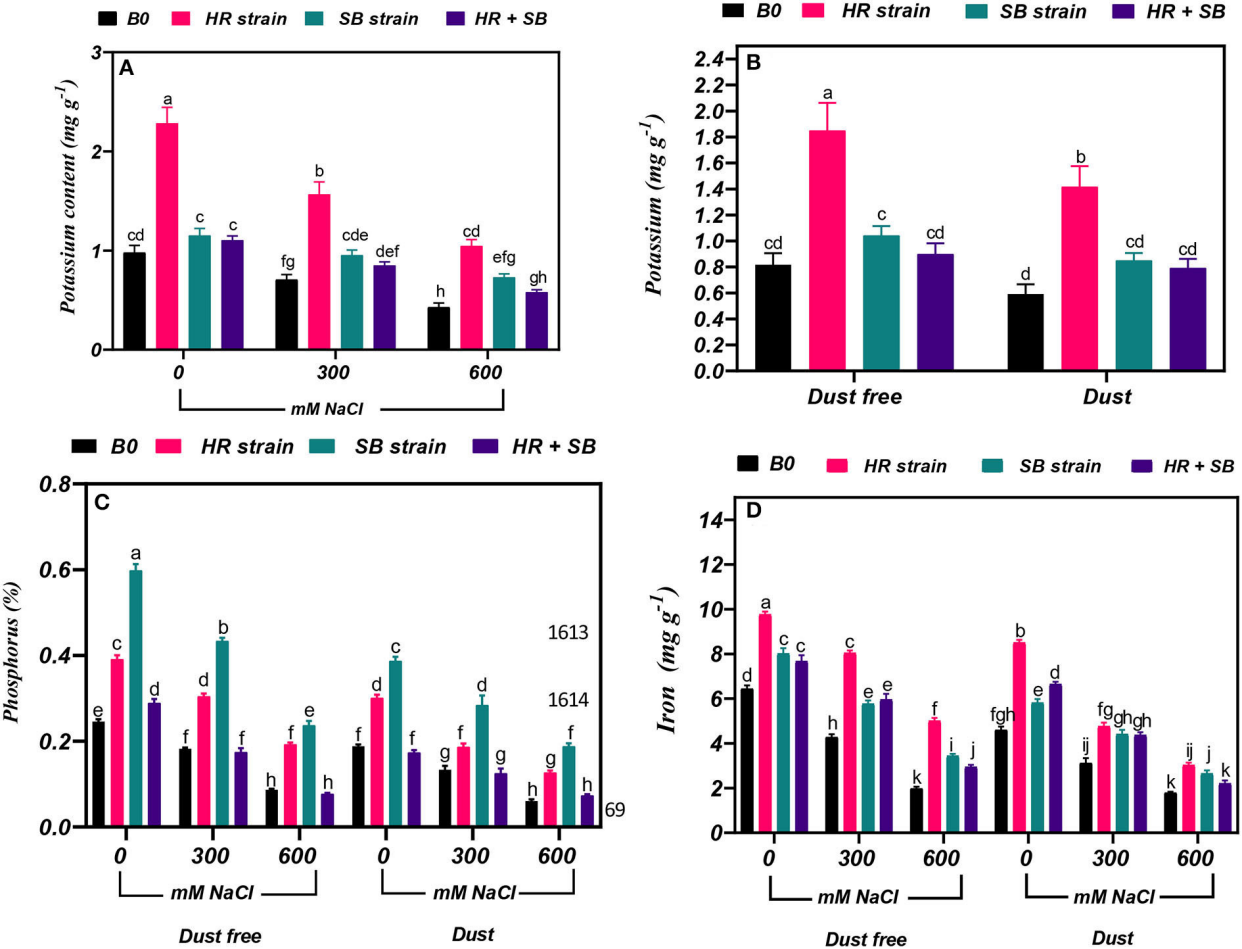
Triple/dual interactions of treatments of halotolerant bacterial strains (*B. pumilus* HR and *Z. halotolerans* SB), salinity levels (irrigation with saline water), and dust on the content of **(A,B)** potassium, **(C)** phosphorus, and **(D)** iron of *Haloxylon aphyllum* seedlings grown under greenhouse conditions for 150 days. Means ± SE (*n* = 3) followed by the same letters are not significantly different according to Duncan's new multiple range test at *P* < 0.05.

Under the bacteria-free and dust-free conditions, potassium content increased by 28 and 56% at the 300- and 600-mM NaCl level, respectively (Figure 5A). By increasing the salinity concentration to the 600-mM NaCl level, the content of potassium was decreased by 49% compared to the control. Under non-saline conditions, the inoculation with the HR strain significantly increased potassium content by 126% under dust-free conditions and by 139% under dust application conditions. Under dust-free conditions, the HR and SB strains increased potassium content by 121 and 35% at the 300-mM NaCl level and by 144 and 70% at the 600-mM NaCl level compared to the control (Figure 5B).

### Effect of bacterial strains on phosphorus content

Under the bacteria-free and dust-free conditions, phosphorus content was decreased by 26 and 65% at the 300- and 600-mM NaCl levels, respectively, and by 29 and 68% under dust application conditions, respectively. The dust treatment alone (without bacteria and salinity) decreased nitrogen content by 23%. The inoculation with the HR strain, SB strain, and the combination of the two strains caused a significant increase in phosphorus content by 59, 143, and 17% under the non-saline and dust-free conditions, while the HR strain and the combination of the two strains increased the content of phosphorus by 59 and 105% under dust application conditions. The HR and SB strains increased the content of phosphorus at the 300-mM NaCl level by 67 and 138%, and by 122 and 173% at the 600-mM NaCl level under dust-free conditions. Also, by applying dust at the 300-mM NaCl level, the HR and SB strains increased the content of phosphorus by 40 and 113%, respectively, and by 110 and 209% at the 600-mM NaCl level compared to the control, respectively (Figure 5C).

### Effect of bacterial strains on iron content

Under the bacteria-free and dust-free conditions, iron content was decreased by 34 and 69% at the 300- and 600-mM NaCl levels, respectively, and by 32 and 61% under dust application conditions, respectively. The dust treatment alone (without bacteria and salinity) decreased iron content by 28%. Under non-saline conditions, the inoculation with the HR strain, SB strain, and the combination of the two strains significantly increased iron content by 52, 24 and 19%, respectively, under dust free conditions and by 85, 27, and 45%, respectively, under dust application conditions. The HR strain, SB strain, and the combination of the two strains increased the content of iron at the 300-mM NaCl level by 88, 35, and 39%, and by 153, 74, and 49% at the 600-mM NaCl level under dust-free conditions. Under dust application conditions, the HR strain, SB strain, and the combination of the two strains increased the content of iron at the 300-mM NaCl level by 53, 42, and 40%, respectively. The HR and SB strains increased the content of iron by 69 and 48% at the 600-mM NaCl level compared to the control, respectively, under dust application conditions (Figure 5D).

### Effect of bacterial strains on calcium content

**Figure 6:**
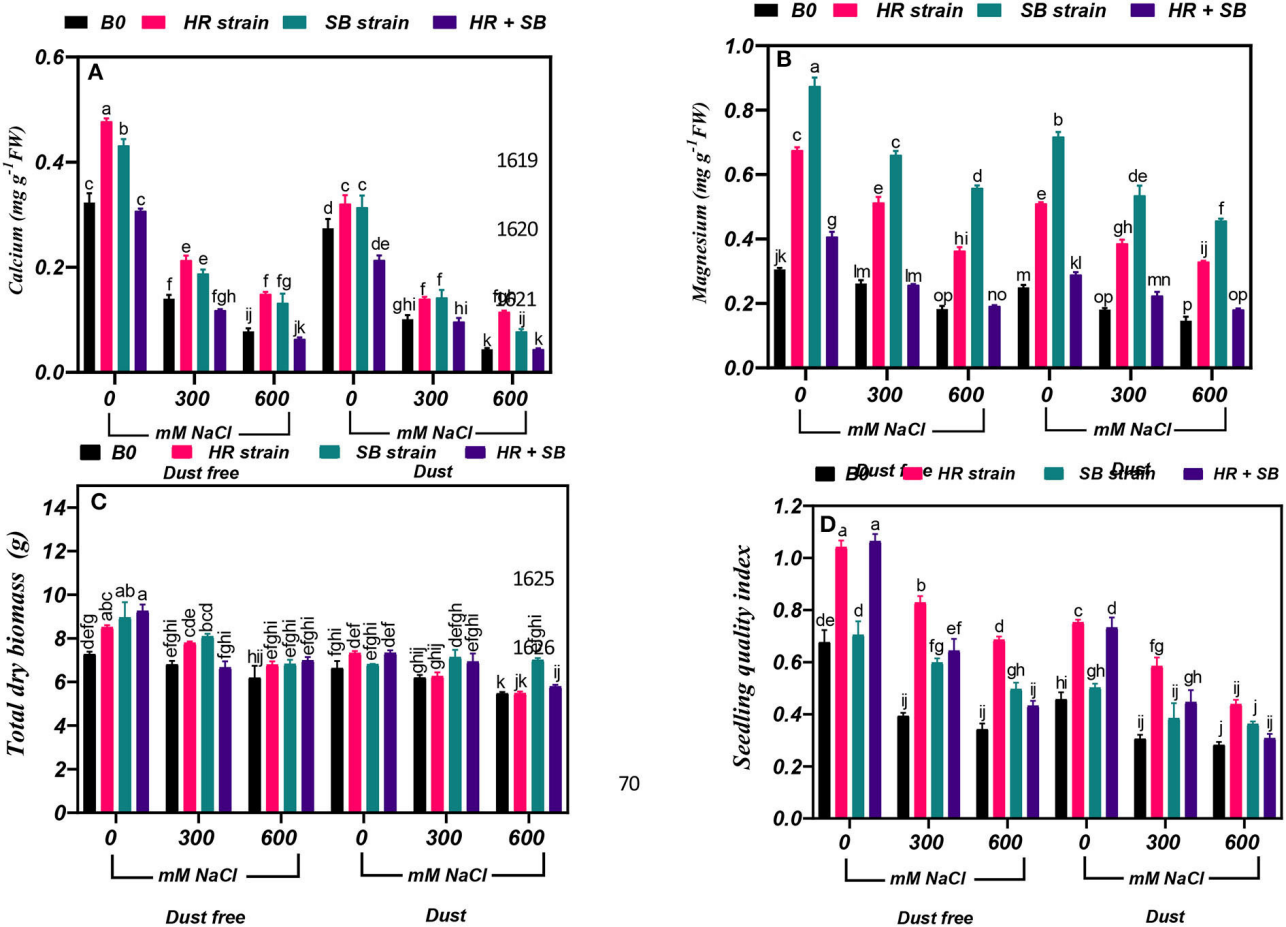
Triple/dual interactions of treatments of halotolerant bacterial strains (*B. pumilus* HR and *Z. halotolerans* SB), salinity levels (irrigation with saline water), and dust on the content of **(A)** calcium, **(B)** magnesium, **(C)** total dry biomass, and **(D)** sapling quality index of *Haloxylon aphyllum* seedlings grown under greenhouse conditions for 150 days. Means ± SE (*n* = 3) followed by the same letters are not significantly different according to Duncan's new multiple range test at *P* < 0.05.

Under the bacteria-free and dust-free conditions, calcium content was decreased by 56 and 76% at the 300- and 600-mM NaCl levels, respectively, and by 63 and 84% under dust application conditions, respectively. The dust treatment alone (without bacteria and salinity) decreased calcium content by 15%. Under non-saline conditions, the inoculation with the HR and SB strains significantly increased calcium content by 48 and 33%, respectively, under dust-free conditions and by 17 and 14%, respectively, under dust application conditions. Under dust-free conditions, the HR and SB strains increased the content of calcium by 52 and 34% at the 300-mM NaCl level, respectively, and by 91 and 69% at the 600-mM NaCl level, respectively. The HR and SB strains also increased the content of calcium by 38 and 40% at the 300-mM NaCl level and by 161 and 77% at the 600-mM NaCl level compared to the control, respectively, under dust application conditions (Figure 6A).

### Effect of bacterial strains on magnesium content

Under the bacteria-free and dust-free conditions, magnesium content was decreased by 14 and 40% at the 300- and 600-mM NaCl levels, respectively, and by 27 and 41% under dust application conditions, respectively. The dust treatment alone (without bacteria and salinity) decreased magnesium content by 18%. Under non-saline conditions, the inoculation with the HR strain, SB strain, and the combination of the two strains significantly increased magnesium content by 121, 168, and 33%, respectively, under dust-free conditions and by 104, 187, and 16%, respectively, under dust application conditions. Under dust-free conditions, the HR and SB strains increased the content of magnesium by 96 and 152% at the 300-mM NaCl level, respectively, and by 99 and 206% at the 600-mM NaCl level, respectively. The HR strain, SB strain, and the combination of the two strains significantly increased magnesium content by 113, 196, and 24% at the 300-mM NaCl level, respectively, under dust application conditions. The HR and SB strains also increased the content of magnesium by 125 and 212% at the 600-mM NaCl level compared to the control, respectively, under dust application conditions (Figure 6B).

### Effect of bacterial strains on total dry biomass

In the absence of bacteria and dust, the amount of total dry biomass at the 600-mM NaCl level was decreased by 18% compared to the control. Under non-saline conditions, the inoculation with the HR strain, SB strain, and the combination of the two strains significantly increased total dry biomass by 17, 23 and 28%, respectively, under dust-free conditions. Also, the SB strain increased the amount of total dry biomass by 19% at the 300-mM NaCl level, and at the 600-mM NaCl level, the HR and SB strains increased total dry biomass by 13% under dust-free conditions. Also, by applying dust at the 600-mM NaCl level, the SB strain increased total dry biomass by 28% compared to the control (Figure 6C).

### Effect of bacterial strains on seedling quality index

Under the bacteria-free and dust-free conditions, seedling quality index was decreased by 42 and 49% at the 300- and 600-mM NaCl levels, respectively, and by 33 and 38% under dust application conditions, respectively. The dust treatment alone (without bacteria and salinity) decreased seedling quality index by 32%. Under non-saline conditions, the inoculation with the HR strain and the combination of the two strains significantly increased seedling quality index by 54 and 57%, respectively, under dust-free conditions and by 65 and 60%, respectively, under dust application conditions. The HR strain, SB strain, and the combination of the two strains significantly increased seedling quality index by 111, 52, and 63% at the 300-mM NaCl level, respectively, under dust application conditions. The HR strain increased seedling quality index by 101% at the 600-mM NaCl level under dust-free conditions, while by applying dust with the HR strain and the combination of the two strains increased seedling quality index by 92 and 46%, respectively, at the 300 mM NaCl level. At the 600-mM NaCl level, the HR strain also increased seedling quality index by 56% compared to the control under dust application conditions (Figure 6D).

### Principal component analysis

**Figure 7:**
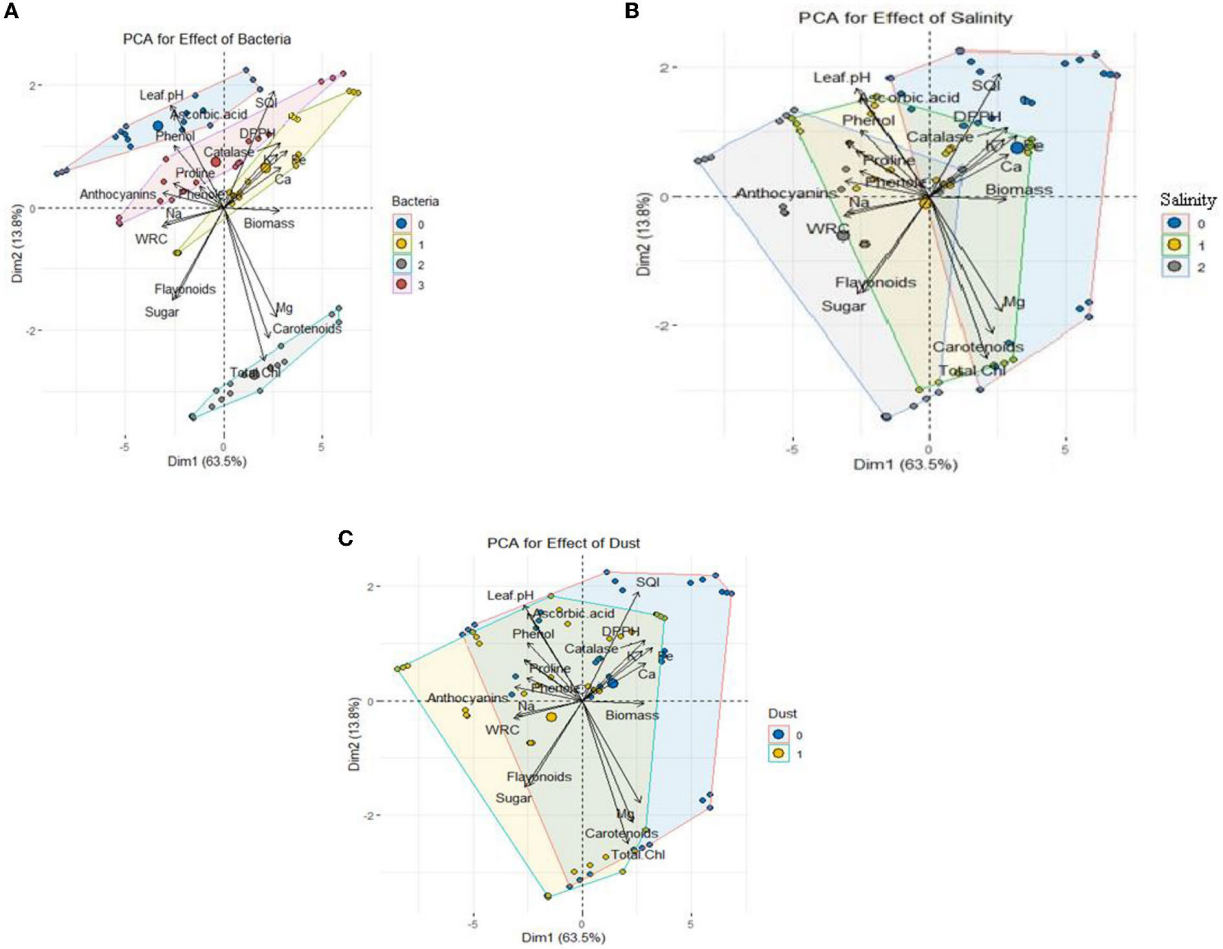
PCA of growth characteristics of *Haloxylon aphyllum* seedlings in response to halotolerant bacterial strain inoculation: **(A)** 0: no bacterial strain, 1: *B. pumilus* HR, 2: *Z. halotolerans* SB, and 3: bacterial consortium (HR and SB); salinity **(B)** 0: control, 1: 300 mM NaCl level, and 2: 600 mM NaCl level; dust **(C)** 0: control and 1: dust application.

According to principal component analysis (PCA) (Figure 7), the measured parameters were classified into three separate clusters based on Pearson correlation (Supplementary Table S1). The parameters positively correlated with each other were placed in a cluster. Cluster 1 includes catalase activity, seedling quality index, 2,2-Diphenyl-1-picrylhydrazyl (DPPH), content of K, Fe, and Ca, and biomass; cluster 2 includes Mg, carotenoids, and total chlorophyll; the third cluster includes leaf acidity, ascorbic acid, phenol, proline, anthocyanin, flavonoid, soluble sugars, and relative water content of the leaves. Figure 7A shows the effect of the bacterial strains on the measured traits. The SB strain showed a positive correlation with cluster 2 including Mg, carotenoids, and total chlorophyll. Also, the presence of the HR strain and the combination of two bacteria increased mineral elements such as Ca, K, and Fe, total dry biomass, and seedling quality index. Figure 7B shows the correlation between salinity and the measured traits. The level of 300 mM NaCl salinity was positively correlated with proline, total phenol, and sodium, and the level of 600 mM NaCl salinity was positively correlated with increased soluble sugars, anthocyanins and flavonoids, and both salinity levels were negatively correlated with calcium, potassium, total dry biomass, and seedling quality index. In other words, these indices were decreased with increase in salinity. The results also showed that the application of dust, similar to salinity, had a negative correlation with calcium, potassium, total dry biomass, and seedling quality index (Figure 7C).

## Discussion

Growing vegetation is an effective way to control wind erosion in drylands, where both dust storms and ephemeral salt lakes (playas) are common (Goudie, 2018). Improved salinity and dust stress tolerance in desert halophytes in these areas can control desertification. Restoration of degraded saline areas was most successful using halophytes inoculated with soil microbiota, including halotolerant PGPR (Qin et al., 2018). Therefore, PGPR are crucial to halophytic plant development, growth, and salt stress tolerance (Komaresofla et al., 2019; Hidri et al., 2022). The optimal salinity for maximum growth of halophytes including the desert halophyte *H. aphyllum* is in the range of 200–400 mM NaCl, and the growth of these plants is significantly decreased if soil salinity is out of this range (Khan et al., 2000). Our findings also indicated that high salinity (600 mM NaCl) significantly reduced the total biomass of *H. aphyllum*. According to Hameed et al. (2012), the growth decline caused by high salinity (600 mM NaCl) may be ascribed to (i) decreased capacity of water absorption by roots, (ii) limited ability of the plant to osmotically adjust, and (iii) toxic ionic effect due to excessive absorption of Na^+^ and Cl^−^, which causes disturbances in nutrient uptake and vital metabolic functions like photosynthesis. Neumann (2011) assumes that growth decrease in response to salt stress might represent an adaptation to boost the odds of surviving long enough to generate seeds.

Although the role of PGPR in alleviating dust stress has not been previously reported and first confirmed in this study, it is well-known that halotolerant PGPR are able to increase salinity-stressed plant biomass by rapidly developing the root system and increasing the ability of plants to absorb nutrients, produce plant hormones, reduce sodium uptake, and increase the expression of genes responsible for resistance to salinity stress (Etesami and Beattie, 2018; Etesami and Maheshwari, 2018). In this study, we also showed that halotolerant bacterial strains with multiple PGP traits, such as production of IAA, siderophore, and ACC deaminase, and phosphate solubilization (Amini Hajiabadi et al., 2021), were able to increase *H. aphyllum* resistance to salinity and dust stress by improving the morpho-physiological, biochemical, and ionomic properties of the plant.

In this study, by increasing salinity level and dust, the content of total chlorophyll of *H. aphyllum* leaf was decreased. In a previous study, increase in dust concentration also induced a significant decrease in the content of total chlorophyll in four plant species (*Fraxinus rotundifolia, Morus alba, Colias caucasica*, and *Melia azedarach*) (Javanmard et al., 2019). A similar trend of reduced total chlorophyll in response to dust pollution has also been reported on Haloxylon aphyllum Bunge (Heydarnezhad, 2014), *Vigna radiata* L. (Alavi, 2015), and *Prunus persica* (Maletsika et al., 2015). Dust deposition on leaf surface may reduce the synthesis of chlorophyll because of shading effects (Setsungnern et al., 2018). Another reason may be related to pigment degradation and inhibition of enzymes essential for biosynthesis of pigments due to incorporation of dust particles into leaf tissues (Lepeduš et al., 2003). Decreased photosynthetic pigments under salinity stress can be due to chlorophyll photooxidation (Sukweenadhi et al., 2018), replacement of Na ion with Mg ion, and decrease in absorption of elements such as K, Fe, and P (Guo et al., 2020). The use of halotolerant bacterial strains used in this study increased the content of total chlorophyll in *H. aphyllum*. The increase in chlorophyll content may be due to the production of siderophore and auxin (e.g., by increasing the root system and improving nitrogen and water uptake) and phosphate solubilization by the bacteria (Etesami and Beattie, 2017; Amini Hajiabadi et al., 2021). For example, siderophore has a strong affinity for bonding with some cations including Fe^3+^, which is an essential element for the formation of chlorophyll (Santoyo et al., 2019). Under the conditions of dust stress, the effect of dust particle application on plant physiology is similar to the effects of drought stress (Rai, 2016). Halotolerant PGPB also reduce the effect of salinity on photosynthesis by increasing water use efficiency in a saline environment and improve photosynthesis and plant growth under saline conditions by increasing chlorophyll concentration (Cheng et al., 2007). In general, the SB strain increased the total chlorophyll content compared to the other bacterial strain, which may be because of the higher auxin production of this strain than the HR strain (Amini Hajiabadi et al., 2021).

The concentrations of anthocyanins, flavonoids, and total phenol, and total DPPH radical scavenging capacity in *H. aphyllum* leaves were increased with increase in salinity and dust levels. In a previous study, when salinity was applied over 24 h to plants of the annual halophyte *Lepidium latifolium*, an increase in total antioxidant capacity, in addition to phenol, ascorbate and flavonoid content, was also observed (Boestfleisch et al., 2014). Synthesis and accumulation of secondary metabolites such as anthocyanins, flavonoids and phenols are the mechanisms of plant tolerance to salinity stress (Wakeel et al., 2011). The association of raised antioxidant capacity with salt tolerance has been demonstrated in a number of salt-tolerant glycophytes and true halophytes (Amor et al., 2005; Alhdad et al., 2013; Boestfleisch et al., 2014). The main functions of these metabolites are mainly associated with their antioxidant role and protection of the photosynthetic system against photooxidation, and they play a protective role in plants under environmental stress (He et al., 2010). These compounds not only eliminate reactive oxygen species (ROS) but also prevent plants from producing more free radicals. Plants can reduce the effect of oxidative stress by producing and accumulating anthocyanins in epidermal layers (Hare and Cress, 1997). Studies show that under environmental stress conditions, an increase in anthocyanin reduces water potential and stomatal conductance (Cartea et al., 2011). Salinity and dust stresses reduce plant access to CO_2_, inhibit carbon fixation, and sequentially reduce molecular oxygen, resulting in overgrowth of ROS species, damage to chloroplast function, and ultimately disruption in photosynthesis process (Hasanuzzaman et al., 2019; Ghanem et al., 2021). The increase and accumulation of secondary metabolites and increase in the content of phenolic compounds under environmental stress act as a signal that can eventually increase the stress tolerance in plants by triggering a chain of reactions (Andersen and Markham, 2005). The long-term effect of alkaline and acid dust particles and their deposition on plant leaves can cause necrosis and increase leaf temperature (Saravana Kumar and Sarala Thambavani, 2012), and this reduces the photosynthetic efficiency and production of plant compounds such as flavonoids (Nanos and Ilias, 2007). In the present study, the bacterial strains used significantly reduced the concentrations of anthocyanins, flavonoids, and total phenol, and total DPPH radical scavenging capacity of *H. aphyllum* leaves at different levels of salinity and dust stress. Decreased concentrations of anthocyanins (Kahil et al., 2017) and total phenol (Al-Garni et al., 2019), and total DPPH radical scavenging capacity (Khan et al., 2020) in plants inoculated with PGPB have been reported in other studies. Soleimanzadeh et al. (2010) showed that increased potassium in sunflower reduced antioxidant activity. Similar results by Kumar et al. (2020) showed that wheat plants inoculated with PGPB increased potassium and decreased antioxidant activity. In the present study, the studied bacteria also increased potassium, which could reduce the activity of antioxidants. These results, which apparently seem to be in contradiction with the assumption that stress resistance in plants is related to more effective antioxidant systems, are an implication of the same positive effect and indicate that inoculated plants felt less stress than uninoculated plants (Carmen and Roberto, 2011).

Leaf pH is an index of detoxification mechanism in plants for improving tolerance capacity against air pollution (Ninave et al., 2001). Ascorbic acid and the pH of *H. aphyllum* leaf extract increased with increase in salinity and dust levels. The penetration of chemical dust particles with an alkaline nature into the cell sap and their conversion to radicals cause an increase in leaf extract pH. High leaf pH also increases the efficiency of conversion from hexose to ascorbic acid (Singare and Talpade, 2013). In the current study, it seems that more probable penetration of alkaline dust particles (pH 7.6) into the leaf tissues, along with growing dust accumulation on the leaf surface, caused increased pH in the leaf extracts.

Production of non-enzymatic antioxidant ascorbic acid is one of the most important defense mechanisms of plants against environmental stresses (as it induces increase in secondary metabolites and antioxidant activity) (Gaafar et al., 2020). To ensure cellular homeostasis and minimize the negative effects of excess ROS, plant cells have evolved a complex antioxidant system, which includes ascorbic acid (Bilska et al., 2019). Ascorbic acid reacts with hydrogen peroxide (H_2_O_2_) and plays a role in neutralizing it and other ROS, leading to protection of carotenoids against various environmental stresses (Chao et al., 2010). Ascorbic acid is also affiliated with chloroplasts in which the effect of oxidative stress on photosynthesis is mitigated (Beltagi, 2008). Ascorbic acid concentration is directly proportional to the pH value of the leaf. Alkaline pH of dust deposited on leaf surface alters cell sap pH toward alkaline and increases the efficiency of the conversion of hexose sugar to ascorbic acid formation (Liu and Ding, 2008; Agbaire and Esiefarienrhe, 2009). The increasing trend in ascorbic acid content as well as leaf extract pH with increase in dust concentration may be attributed to the positive relationship between the two parameters (Rai and Panda, 2014). In the present study, the bacterial strains significantly reduced the concentration of leaf ascorbic acid and pH of the leaf extract at different levels of salinity and dust stress, among which the SB strain had the most effect. It can be concluded that PGPB reduce leaf pH by producing organic acids and amino acids and secreting substances such as protons (Gadd, 2004), which in turn reduce the amount of ascorbic acid. Decreased ascorbic acid concentration in stressed plants inoculated with PGPB has been reported in other studies (Foryer and Noctor, 2000).

The results of this study also showed that with increase in salinity and dust application in *H. aphyllum* species, the content of proline increased. Proline plays an essential role in osmotic regulation and reduction of sodium toxicity in plants (Saddiq et al., 2020). Proline accumulation has a positive and direct relationship with increased resistance to dehydration under salinity and drought stresses in plants (Saneoka et al., 2004). Under salinity stress, proline and catalase activity have antioxidant roles and are used to protect cell structures and the structure of macromolecules and scavenge free radicals (Hafez et al., 2020; Abdelaal et al., 2021). Proline, as an important source of carbon and nitrogen, also plays a vital role in preventing the inactivation of proteins (Xiao et al., 2009). An increase in proline content under salinity stress (Ghanem et al., 2021; Mohamed et al., 2021) can be due to reduction in its conversion to glutamate, reducing its use in protein synthesis (Claussen, 2005). The amino acid proline also plays a very important role in plants and protects them from various stresses, including dust (Gupta et al., 2015). Studies show that under dust stress conditions, proline production in plants is also increased (Yaghmaei et al., 2020). The increase in proline content by applying dust indicates the occurrence of stress, which can be justified because of the reduction of available plant light and disruption of gas exchange due to deposition of dust on plant surfaces. The inoculation of *H*. *aphyllum* with bacterial strains in this study reduced the proline content in this halophytic species, among which the HR strain had the greatest effect on reducing proline content. It is known that PGPB alleviate environmental stress by increasing the availability of potassium and its uptake by plants, which in turn acidify the rhizosphere environment by increasing water uptake by auxin production and increasing root volume by regulating cellular osmotic balance, eliminating active oxygen forms, and protecting proteins in plants. All these mechanisms can justify the reduction in production of antioxidants (Etesami and Maheshwari, 2018; Etesami and Glick, 2020). Decreased proline, due to stress-induced damage repair, can provide energy (ATP required for oxidative phosphorylation) from proline breakdown. Decreased proline content in stressed plants inoculated with PGPB has been reported in other studies (Abd_Allah et al., 2018).

The results of this study also showed that with increase in salinity and dust application in *H. aphyllum* species, catalase activity was increased. Omar et al. (2009) also reported that salinity led to a significant increase of catalase and peroxidase (POD) activities in salt-stressed leaves of two barley cultivars differing in salinity tolerance. Under normal growth conditions, the production of ROS in cells is low, whereas during stress the rate of production is enhanced. ROS accumulation during stress results from the imbalance between production and scavenging of ROS (Etesami and Maheshwari, 2018). Plants are armed with an antioxidant defense system to protect themselves against the harmful effects of ROS. Despite this antioxidant defense system, a decline in plant yield has been reported under oxidative stress conditions, which indicates that environmental stresses (e.g., salinity, drought, etc.) have altered the activity of enzymes such as CAT and POD in stressed plants (Paul and Lade, 2014). For detoxification of excess ROS in plants, the overall balance between different antioxidants is crucial for determining the steady-state level of superoxide radicals and hydrogen peroxide, and has to be tightly controlled (Mittler, 2002). There is considerable evidence that salt-tolerant PGPR-inoculated plants can survive under oxidative conditions by manipulating the antioxidant defense system (Etesami and Glick, 2020). As an example, plants inoculated with PGPR (e.g., *Enterobacter* spp. and *Bacillus* spp.) showed higher activities of antioxidant enzymes (e.g., SOD and CAT) than non-inoculated plants under saline stress conditions (Carmen and Roberto, 2011). PGPR can alleviate oxidative degradation in salt stressed plants by interference in the regulation of antioxidant defense (Etesami and Glick, 2020).

With increase in salinity and dust, the relative water content (RWC) of *H. aphyllum* was decreased, which is in line with earlier studies on other plants (Maletsika et al., 2015; Karami et al., 2017). For example, in a previous study, increase in dust concentration also induced a significant decrease in the RWC four species (*F. rotundifolia, M. alba, C. caucasica*, and *M. azedarach*) (Javanmard et al., 2019). Leaf RWC is a good indicator of water status and content of water in plant organs; it is suggested as a resistance index to withstand environmental stress and shows the ability of that plant to retain water under different stress conditions (Ritchie et al., 1990). The decrease in RWC under salinity conditions is due to limited plant access to water for the cell development process and indicates a decrease in cell turbidity. Low RWC may be due to the effect of dust pollutant on leaf transpiration, since the crust formed by dust deposition on leaves can lead to blockage of the stomata and reduced transpiration (Zia-Khan et al., 2014; Masoud et al., 2019). Also, the reaction of dust particles with the cell membrane results in foliar injury and higher membrane permeability in dusted leaves, which may be another reason for low RWC value (Yaghmaei et al., 2020). Dust particles also cause loss of water and soluble nutrients in plants by increasing the permeability of plant cells and eventually causing premature aging of leaves. Therefore, plants with a higher RWC are more resistant to dust (Loganathan and Ilyas, 2012; Taheri Analojeh et al., 2016). The inoculation with bacterial strains in this study caused an increase in the RWC of *H. aphyllum*. The inoculation with the HR strain and combination of the two strains (HR + SB) had the greatest effect on increase in RWC. One of the reasons for this increase in inoculated plants could be the production of growth hormones such as auxin and, thus, increase in the length and weight of roots compared to the control plants under stress conditions (Noori et al., 2018; Ansari et al., 2019). These bacteria may also decrease the production of abscisic acid (Naz et al., 2009); in this way, they reduce the negative effects of stress on stomatal conductance, photosynthesis, and plant sensitivity to water deficiency (Etesami and Beattie, 2018). It seems that the plants inoculated with the bacterial strains have the ability to change the structure of the lateral root system and increase RWC (Bertrand et al., 2015; Shirmohammadi et al., 2020). Increased leaf RWC under stress in plants inoculated with PGPB has also been reported in other studies (Ilyas et al., 2020; Shilev, 2020). In general, an increase in the number of lateral roots and root hairs causes addition of root surfaces available for nutrient and water uptake. Higher water and nutrient uptake by inoculated roots causes an improved water status of plants, which in turn could be the main factor enhancing plant growth (Etesami and Maheshwari, 2018).

With increase in salinity and dust, the content of malondialdehyde (MDA) in *H. aphyllum* was increased. Soil salinity increases the cellular levels of ROS that leads to lipid peroxidation of membranes (Gill and Tuteja, 2010), and increases the content of the biomarker MDA (Das and Roychoudhury, 2014) and relative electric conductivity (Noctor et al., 2014). In previous studies, increased levels of MDA have been shown in plants under salinity stress (Ghanem et al., 2021) and dust conditions (Davey et al., 2005). In this study, the inoculation with bacterial strains under stress conditions reduced MDA in *H. aphyllum*, as the inoculation with the HR strain had the greatest effect on reduction in the amount of MDA in this plant. The reduction in MDA content suggests that inoculation better protects the plants from the imposed oxidative stress caused by NaCl. Our results are consistent with previous studies on the coastal halophyte *Ligustrum sinense* and *Salicornia* sp. inoculated with *Glutamicibacter halophytocola* KLBMP 5180 and *Staphylococcus* sp. R11, respectively, and treated with salt (Qin et al., 2018; Komaresofla et al., 2019). In a previous study, the halophyte *Suaeda fruticosa*-inoculated plants with *Glutamicibacter* sp. and *Pseudomonas* sp. exhibited lower MDA contents than the non-inoculated plants under saline conditions (Hidri et al., 2022). The ameliorative effect on MDA accumulation in plants inoculated with PGPR may also be related to the higher accumulation of nitrogen-containing compounds such as proline (Hidri et al., 2016), which is involved in stabilization of subcellular structures (membrane and proteins), scavenges free radicals, and buffers cellular redox potential under stress conditions (Kishor et al., 2005).

The survival and productivity of crop plants exposed to environmental stresses (salinity and dust) are dependent on their ability to develop adaptive mechanisms to avoid or tolerate stress (Munns and Tester, 2008). Increasing evidence suggests that the mineral nutritional status of plants greatly affects their ability to adapt to adverse environmental conditions, in particular to abiotic stress factors. Impairment of the mineral nutrition status of plants exacerbates the adverse effects of salinity and dust stresses, and exogenous addition of high levels of macro/micronutrients can alleviate the adverse effects of stress on plant growth (Etesami and Maheshwari, 2018). In this study, with increase in salinity and dust, the content of Na ions was increased and the amount of K ions was decreased. To survive under salt stress conditions, it is essential for plants to maintain lower Na^+^ and Cl^−^ contents in their tissues (Esechie et al., 2002). Thus, controlling Na^+^ homeostasis is critical to maintain normal plant growth during salt and dust stress. Because of their physicochemical similarities, Na and K ions compete for transferring into plants. Under saline stress conditions, plants need more K because a large amount of K is consumed during protein synthesis and enzyme activity under these conditions (Marschner, 1995). Sodium is unable to perform other physiological and biochemical functions of K other than to supply turgor pressure. Therefore, under salinity stress, energy transfer, protein production, enzymatic-activity, motility of stomatal protective cells, and photosynthesis that require K are impaired and lead to diminished plant growth (Rubio et al., 2020). Numerous studies have reported an inverse relationship between Na ion accumulation and photosynthesis in plants (Shilev, 2020). Under the conditions of dust stress, the effect of dust particle application in plant physiology is similar to the effects of drought stress (Rai, 2016). The increase in Na ion under dust stress, such as drought stress, is probably due to the greater uptake of this ion by the roots and its further discharge from the xylem vessels to leaves. The inoculation with the bacterial strains used in this study under stress condition decreased Na ions and increased K ions in *H. aphyllum*, as the inoculation with the HR strain had the greatest effect on reducing Na ion content and increasing K ion content, strongly suggesting an efficient inhibition of translocation. This could be achieved by tissue-specific regulation of HKT1, a plasma membrane Na^+^ uniporter, or by promoting biofilm formation on root surfaces, thus restricting Na^+^ influx into roots (Zhang et al., 2008; Gerhardt et al., 2017). Talaat et al. (2015) suggested that this may be due to the dilution effect associated with plant growth improvement and the enhanced availability of P in the rhizosphere that reduced Na^+^ uptake under saline conditions. In a previous study, PGPR inoculation decreased Na^+^ content in shoots of the halophyte *Suaeda fruticosa* under 600-mM NaCl stress conditions (Hidri et al., 2022). Limited Na^+^ influx is thought to be a salt tolerance mechanism that protects the photosynthetic apparatus of H. *aphyllum* from salt damage by decreasing Na^+^ ion translocation to the aerial part (Islam et al., 2016).

Potassium is the pivotal inorganic ion that participates in cellular osmotic adjustment, and absorption of K^+^ improves the water uptake capacity of plants and consequently alleviates salt-induced osmotic stress (Tallapragada and Bagyaraj, 2017). In this study, the bacterial strains increased the concentration of K in *H. aphyllum* under stress conditions, and this effect may be involved in the maintenance of turgor pressure and mitigation of oxidative stress imposed by excessive salinity (Upadhyaya et al., 2011). The function of bacterial strains in the present study in increasing K concentration is consistent with the results of other studies in this field (Pan et al., 2019; Baek et al., 2020). For example, in a previous study, *Pseudomonas* sp. inoculation also enhanced potassium contents in the shoots of the halophyte *Suaeda fruticose*. PGPB increase the absorbable form of K ions in the rhizosphere by affecting K-bearing minerals (Etesami et al., 2017) and give the plant the opportunity to increase ionic balance by boosting the flow of K ions to the aerial part and transferring Na^+^ to the roots (Wang et al., 2016). In this regard, the ability to produce exopolysaccharide biopolymers by PGPB (Radhakrishnan et al., 2017), by trapping Na ions, can also act as a barrier to excess Na entering plant roots.

In this study, with increase in salinity and dust, leaf phosphorus concentration was decreased. In particular, it is known that salt stress causes reduction in P accumulation in plants, which developed P-deficiency symptoms (Carmen and Roberto, 2011). The reduction in P availability in saline soils was suggested to be a result of ionic strength effects that reduce the activity of phosphate and tight control of P concentrations by sorption processes and by low solubility of Ca-P minerals (Etesami and Maheshwari, 2018). Phosphorus is one of the most important nutrients needed for plant growth and development. This element helps to store and transfer energy in the process of photosynthesis and is essential for cell division and RNA and DNA formation (Etesami, 2020). It is well-known that PGPB increase the availability of phosphorus to plants under environmental stress by production of phosphatase, phytase enzymes, and organic acids (Etesami, 2020). By lowering the pH of the rhizosphere by releasing organic acids, these bacteria dissolve insoluble phosphorus minerals and convert them into an absorbable form for plants. Increased phosphorus concentration in plants inoculated with PGPB under environmental stress has also been reported in other studies (Karimzadeh et al., 2020; Shirmohammadi et al., 2020).

In this study, with increase in salinity level and dust, leaf Fe content was decreased. Reduction in Fe uptake under saline conditions has been expressed as one of the effective factors in reducing chlorophyll and the photosynthesis process (Karimi et al., 2009). Decreased content of Fe due to dust stress in plants has also been reported (Mutlu et al., 2013). In this study, the bacterial strains in all the three cases increased the content of iron in *H. aphyllum* species, among which inoculation with the HR strain had the greatest effect on increase in iron level. When a plant is deficient in Fe, the bacteria associated with the plant secrete a low-molecular-weight organic compound called siderophore that has a strong affinity for being linked to some cations, including Fe (III) (Ahmad et al., 2008; Santoyo et al., 2019). The plant can use this bacterial siderophore to supply Fe. Increased Fe concentration under environmental stress caused by PGPB has also been reported in other studies (Kotasthane et al., 2017; Karimzadeh et al., 2020).

In this study, with increase in salinity level and dust application, leaf Mg and Ca concentrations were decreased. The reduction in the content of these elements in *H. aphyllum* leaves may be due to competition between these nutrients and Na ions. Impaired absorption of Mg in leaves of the *H. aphyllum* plant can probably occur because of the interaction of Na and Mg ions. It is known that the amount of Mg absorption decreases because of the high concentration of Na ions under salinity stress (Karimi et al., 2009). Since the most important role of Mg is to participate in the structure of chlorophyll, environmental stresses, including salinity stress, cause degradation and decrease in chlorophyll concentration in plants. In the present study, the leaf Mg concentration of *H. aphyllum* inoculated with all the bacteria increased significantly under salinity and dust stress. The SB strain had the greatest effect on increase in the leaf Mg concentration of *H. aphyllum*. PGPB are able to increase plant tolerance to stress by rapidly developing the root system and increasing the ability of the plant to absorb nutrients such as Mg (Alkahtani et al., 2020).

Higher root Ca^2+^ content under salt and dust stress also appears to partly contribute to the improved growth of the investigated species. Interestingly, the inoculation with bacterial strains increased Ca^2+^ in the shoots of *H. aphyllum* compared to the non-inoculated stressed plants, supporting a possible PGPR inoculation-mediated Ca^2+^ uptake for Na^+^ homeostasis at the cellular/tissue level. In a previous study, *Pseudomonas* sp. increased Ca^2+^ in shoots of *H. aphyllum* under salinity and dust conditions (Hidri et al., 2022). By increasing the content of Ca ions, plants are protected from the toxicity of sodium chloride by moving the Ca-related membrane and reducing the absorption of Na ions and transferring it to shoots (Grattan and Grieve, 1998). Thus, we hypothesize that utilization of inorganic ions (potassium, iron, magnesium, and calcium) in the plants inoculated with *B. pumilus* HR and *Z. halotolerans* SB relieves the physiological drought under dust and salt-induced osmotic stress, strengthens the osmotic adjustment capacity, and allows for the allocation of energy to be used for growth to a certain extent.

In this study, with increase in salinity concentration and dust application, the content of total dry biomass and seedling quality index of *H. aphyllum* were decreased. The present investigation showed that halotolerant PGPR inoculation significantly increased the total biomass of *H. aphyllum* plants when compared to dust and salt-stressed non-inoculated plants under dust and salinity stress. Similar improvement in plant growth by PGPR has been reported with other halophytes such as *Limonium sinense, Salicornia* sp., *Suaeda fruticose, Elaeagnus aangustifolia* L., and *Puccinellia tenuiflora* (Qin et al., 2018; Komaresofla et al., 2019; Bueno and Cordovilla, 2021; Hidri et al., 2022). Roots are the first “bar of defense” when growing in a saline soil, and root system indicators are often used to quantify the acquisition capacity of water and nutrients in plants (Brundrett, 1991). Interestingly, *B. pumilus* HR and *Z. halotolerans* SB inoculation led to a higher root dry weight compared to non-inoculated stressed plants and alleviated significantly the biomass due to salinity and dust, which reflects the promotive effect of these bacterial strains under stress conditions. This also corroborates previous studies highlighting that PGPR colonization positively modulated the root-system architecture and growth under salt stress conditions (Egamberdieva et al., 2017; El-Esawi et al., 2018).

It is known that leaf surfaces covered with dust particles receive less light for the process of photosynthesis, which leads to closing of the leaf stomata and thus reduction in stomatal conductance (Lepeduš et al., 2003). Decreased stomatal conductance in plants ultimately has a negative effect on biomass production and plant yield. Dust stress has a significant effect on root and shoot dry weight by reducing leaf water content and generally reduces dry matter production in all plant organs (Bauerle et al., 2007). Decreases in total plant biomass due to dust stress have also been reported in other studies (Yaghmaei et al., 2020). In the present study, dry matter production was significantly increased in the *H. aphyllum* inoculated with bacterial strains under dust stress. The SB strain had the greatest effect on the increase in this parameter. It seems that the bacterial strains increased the availability of water and nutrients to *H. aphyllum* under dust stress by increasing root growth, and as a result, increasing plant growth and finally the dry biomass index of the whole plant.

Seedling quality index in all bacterial treatments was improved at the level of stress treatments compared to the control. PGPR are known to increase the root growth of the plant by lowering the ethylene concentration of ACC deaminase under stress conditions (Del Carmen Orozco-Mosqueda et al., 2020). The growth improvement recorded in inoculated *H. aphyllum* plants exposed to salt and dust stress may be due to the various PGP characteristics of the inoculated strains. *B. pumilus* HR and *Z. halotolerans* SB have been reported for solubilization of phosphorus and production of plant growth regulators (like auxin), ACC deaminase, and siderophore (Amini Hajiabadi et al., 2021). Inoculation with both bacterial strains and their combination increased the seedling quality index of *H. aphyllum* plants under dust and NaCl stress, which is consistent with previous findings on the halophyte grass *Puccinellia tenuiflora*, where inoculation with *Bacillus subtilis* GB03, *Glutamicibacter* sp., and *Pseudomonas* sp. was found to affect the biomass of these plants (Bueno and Cordovilla, 2021; Hidri et al., 2022) and the compositional structure of seedling quality index (e.g., total dry weight and root-to-shoot ratio) of other plants (Bhatt et al., 2020). In general, the study clearly reveals that the application of halotolerant bacterial strains may be a cost-effective and ecological sustainable method to improve the tolerance of *H. aphyllum* plants grown in desert areas under salinity stress and dust stress, which was confirmed in this study for first time.

## Conclusions

The results of this study showed that the halotolerant bacterial strains improved the morphological, physiological, and biochemical characteristics of *H. aphyllum* seedlings under salinity and dust stress compared to the control treatment. The studied bacteria not only improved the plant growth conditions under salinity stress but also alleviated the negative effects of dust on this plant. It can be concluded that the rhizosphere of halophytic plants is a good source for the isolation of halotolerant PGPR; and that they can be promising in creating vegetation on the edge of deserts and increasing carbon sequestration. However, it is necessary to conduct additional field research in the habitat of this desert species to prove the function and effectiveness of the halotolerant bacterial strains as a suitable biofertilizer for dealing with salinity stress and dust conditions in the future.

## Data availability statement

The datasets presented in this study can be found in online repositories. The names of the repository/repositories and accession number(s) can be found in the article/Supplementary material.

## Author contributions

All authors listed have made a substantial, direct, and intellectual contribution to the work and approved it for publication.

## Conflict of interest

The authors declare that the research was conducted in the absence of any commercial or financial relationships that could be construed as a potential conflict of interest.

## Publisher's note

All claims expressed in this article are solely those of the authors and do not necessarily represent those of their affiliated organizations, or those of the publisher, the editors and the reviewers. Any product that may be evaluated in this article, or claim that may be made by its manufacturer, is not guaranteed or endorsed by the publisher.
