# Supplementary material for: Improved salinity and dust stress tolerance in the desert halophyte Haloxylon aphyllum by halotolerant plant growth-promoting rhizobacteria
Source: Front Plant Sci. 2022 Aug 3;13:948260. doi: 10.3389/fpls.2022.948260 (PMC9382590; doi:10.3389/fpls.2022.948260)
Supplement: Supplementary file 1 [file Table_1.DOC]

| Table S1: The correlations among the measured traits in this study | | | | | | | | | | | | | | | | | | | | |
| --- | --- | --- | --- | --- | --- | --- | --- | --- | --- | --- | --- | --- | --- | --- | --- | --- | --- | --- | --- | --- |
| Variables | Chlt | Car | Anth | Ascor | Flav | Phenol | Prol | Sugars | pH | Cata | RWC | DPPH | MDA | Na | K | P | Ca | Mg | Biomass | SQI |
| Chlt | 1 |  |  |  |  |  |  |  |  |  |  |  |  |  |  |  |  |  |  |  |
| Car | .838^**^ | 1 |  |  |  |  |  |  |  |  |  |  |  |  |  |  |  |  |  |  |
| Anth | -.564^**^ | -.553^**^ |  |  |  |  |  |  |  |  |  |  |  |  |  |  |  |  |  |  |
| Ascor | -.827^**^ | -.603^**^ | .811^**^ | 1 |  |  |  |  |  |  |  |  |  |  |  |  |  |  |  |  |
| Flav | -.157 | -.113 | .718^**^ | .541^**^ | 1 |  |  |  |  |  |  |  |  |  |  |  |  |  |  |  |
| Phenol | -.520^**^ | -.577^**^ | .736^**^ | .581^**^ | .256^*^ | 1 |  |  |  |  |  |  |  |  |  |  |  |  |  |  |
| Prol | -.711^**^ | -.635^**^ | .669^**^ | .754^**^ | .608^**^ | .332^**^ | 1 |  |  |  |  |  |  |  |  |  |  |  |  |  |
| Sugars | -.593^**^ | -.712^**^ | .559^**^ | .472^**^ | .402^**^ | .331^**^ | .721^**^ | 1 |  |  |  |  |  |  |  |  |  |  |  |  |
| PH | -.129 | -.299^*^ | .691^**^ | .307^**^ | .641^**^ | .420^**^ | .469^**^ | .540^**^ | 1 |  |  |  |  |  |  |  |  |  |  |  |
| Cata | -.777^**^ | -.681^**^ | .846^**^ | .896^**^ | .431^**^ | .810^**^ | .653^**^ | .475^**^ | .332^**^ | 1 |  |  |  |  |  |  |  |  |  |  |
| RWC | .265^*^ | .382^**^ | -.677^**^ | -.406^**^ | -.613^**^ | -.638^**^ | -.456^**^ | -.548^**^ | -.720^**^ | -.477^**^ | 1 |  |  |  |  |  |  |  |  |  |
| DPHH | -.501^**^ | -.584^**^ | .734^**^ | .591^**^ | .663^**^ | .587^**^ | .787^**^ | .658^**^ | .720^**^ | .618^**^ | -.798^**^ | 1 |  |  |  |  |  |  |  |  |
| MDA | -.619^**^ | -.659^**^ | .886^**^ | .774^**^ | .546^**^ | .845^**^ | .676^**^ | .549^**^ | .564^**^ | .900^**^ | -.640^**^ | .756^**^ | 1 |  |  |  |  |  |  |  |
| Na | -.364^**^ | -.556^**^ | .780^**^ | .478^**^ | .547^**^ | .795^**^ | .509^**^ | .526^**^ | .735^**^ | .659^**^ | -.787^**^ | .845^**^ | .837^**^ | 1 |  |  |  |  |  |  |
| K | .213 | .275^*^ | -.726^**^ | -.439^**^ | -.613^**^ | -.715^**^ | -.406^**^ | -.380^**^ | -.633^**^ | -.600^**^ | .748^**^ | -.693^**^ | -.687^**^ | -.767^**^ | 1 |  |  |  |  |  |
| P | .713^**^ | .865^**^ | -.669^**^ | -.557^**^ | -.338^**^ | -.678^**^ | -.645^**^ | -.737^**^ | -.543^**^ | -.681^**^ | .601^**^ | -.722^**^ | -.714^**^ | -.725^**^ | .586^**^ | 1 |  |  |  |  |
| Ca | .323^**^ | .549^**^ | -.627^**^ | -.342^**^ | -.553^**^ | -.564^**^ | -.584^**^ | -.614^**^ | -.727^**^ | -.462^**^ | .733^**^ | -.819^**^ | -.659^**^ | -.770^**^ | .754^**^ | .801^**^ | 1 |  |  |  |
| Mg | .806^**^ | .888^**^ | -.673^**^ | -.660^**^ | -.214 | -.779^**^ | -.598^**^ | -.638^**^ | -.369^**^ | -.795^**^ | .505^**^ | -.629^**^ | -.757^**^ | -.654^**^ | .569^**^ | .937^**^ | .684^**^ | 1 |  |  |
| Biomass | .556^**^ | .653^**^ | -.585^**^ | -.432^**^ | -.411^**^ | -.544^**^ | -.600^**^ | -.776^**^ | -.617^**^ | -.498^**^ | .720^**^ | -.775^**^ | -.600^**^ | -.691^**^ | .558^**^ | .790^**^ | .730^**^ | .676^**^ | 1 |  |
| SQI | .088 | .051 | -.655^**^ | -.374^**^ | -.769^**^ | -.456^**^ | -.405^**^ | -.409^**^ | -.712^**^ | -.374^**^ | .819^**^ | -.687^**^ | -.534^**^ | -.642^**^ | .791^**^ | .374^**^ | .653^**^ | .263^*^ | .585^**^ | 1 |

Abbreviations: Chlt, total chlorophyll; Car, carotenoids; Anth, anthocyanin; Ascor, ascorbic acid; Flav, flavonoid; Pro, proline; Cata, catalase; WRC, relative water content; DPPH, 2,2-Diphenyl-1-picrylhydrazyl radical scavenging capacity; MDA, malondialdehyde; and SQI, seedling quality index.

** and *, correlation is significant at 0.01 level (2-tailed) and 0.05 level (2-tailed), respectively.
